# Supplementary material for: Gut microbiota induces high platelet response in patients with ST segment elevation myocardial infarction after ticagrelor treatment
Source: eLife. 2022 Mar 8;11:e70240. doi: 10.7554/eLife.70240 (PMC8903831; doi:10.7554/eLife.70240)
Supplement: Figure 1—source data 5. — NPR, normal platelet reactivity; HPR, high platelet reactivity; MI, myocardial infarction; PCI, percutaneous coronary intervention; CABG, coronary artery bypass grafting; ACE, angiotensin-converting enzyme; PPI, proton pump inhibitor; WBC, white blood cell; FPG, fasting plasma glucose; TC, serum total cholesterol; TG, serum triglyceride; LDL-C, serum low-density lipoprotein cholesterol; HDL-C, serum high-density lipoprotein cholesterol. Data are expressed as the mean ± SD (standard deviation) or n (%), p-value determined by unpaired t-test for continuous variables or Fisher’s exact test for proportions. [file elife-70240-fig1-data5.docx]

| **Variable** | **NPR**  **(n=90)** | **HPR**  **(n=65)** | **P value** |
| --- | --- | --- | --- |
| Age, y | 59±13 | 61±11 | 0.171 |
| Male, n(%) | 78 (87) | 50 (77) | 0.115 |
| Weight, kg | 74±12 | 70±16 | 0.077 |
| **Medical history, n(%)** |  |  |  |
| Smoking | 58 (68) | 34 (56) | 0.123 |
| Hypertension | 45 (51) | 40 (62) | 0.164 |
| Diabetes | 19 (22) | 18 (28) | 0.397 |
| Prior MI | 15 (17) | 10 (16) | 0.809 |
| Prior PCI | 9 (10) | 7 (11) | 0.919 |
| Prior CABG | 0 (0) | 1 (2) | 0.224 |
| **Medications, n(%)** |  |  |  |
| Statins | 90 (100) | 64 (100) | NA |
| ACE inhibitors | 73 (81) | 46 (73) | 0.236 |
| Beta-blocker | 64 (71) | 50 (78) | 0.328 |
| Nitrate | 33 (37) | 20 (32) | 0.529 |
| PPI | 51 (57) | 40 (62) | 0.468 |
| Diuretic | 35 (39) | 20 (31) | 0.330 |
| AngiotensinⅡblocker | 33 (37) | 17 (27) | 0.187 |
| Insulin | 6 (7) | 9 (14) | 0.127 |
| Hypoglycemics | 10 (11) | 10 (15) | 0.412 |
| **Laboratory data** |  |  |  |
| WBC (10^9^/L) | 10.5±2.5 | 10.4±2.4 | 0.772 |
| Platelets (10^9^/L) | 206±47 | 203±53 | 0.697 |
| Hemoglobin (g/L) | 141±15 | 138±16 | 0.234 |
| FPG (mmol/L) | 8.0±3.9 | 8.2±3.8 | 0.237 |
| Cholesterol (mmol/L) | 4.9±1.0 | 5.1±1.2 | 0.448 |
| Triglyceride (mmol/L) | 1.8±1.0 | 1.6±0.9 | 0.258 |
| LDL-C (mmol/L) | 2.9±0.7 | 3.0±0.9 | 0.801 |
| HDL-C (mmol/L) | 1.0±0.4 | 1.1±0.4 | 0.127 |
| Bilirubin (umol/L) | 17.4±8.8 | 15.5±5.7 | 0.129 |
| Creatinine (umol/L) | 77.4±34.8 | 75.5±25.5 | 0.709 |
| Urea nitrogen (mmol/L) | 6.1±2.6 | 6.0±2.2 | 0.953 |

**Figure 1-source data 1. Demographic and clinical characteristics.**

NPR normal platelet reactivity; HPR, high platelet reactivity; MI, myocardial infarction; PCI, percutaneous coronary intervention; CABG, coronary artery bypass grafting; ACE, angiotensin-converting enzyme; PPI, proton pump inhibitor; WBC, white blood cell; FPG, fasting plasma glucose. TC, serum total cholesterol; TG, serum triglyceride; LDL-C, serum low-density lipoprotein cholesterol; HDL-C, serum high-density lipoprotein cholesterol. Data are expressed as the mean ± SD or n (%), *P* value determined by unpaired t-test for continuous variables or Fisher’s exact test for proportions.
