# Supplementary material for: Gut microbiota induces high platelet response in patients with ST segment elevation myocardial infarction after ticagrelor treatment
Source: eLife. 2022 Mar 8;11:e70240. doi: 10.7554/eLife.70240 (PMC8903831; doi:10.7554/eLife.70240)
Supplement: Table 1—source data 1. — MAF, miner allele frequence. [file elife-70240-table1-data1.docx]

| **SNP** | **Chromosome** | **Position** | **MAF** |
| --- | --- | --- | --- |
| rs1922242 | chr7 | 87173667 | 0.396 |
| rs2235048 | chr7 | 87138511 | 0.399 |
| rs1045642 | chr7 | 87138645 | 0.397 |

**Table 1-source data 1 *ABCB1* tagSNPs from the HapMap database**

MAF, miner allele frequence.

| **SNP** | **Primer（5’→3’）** | **Annealing temperature(℃)** | **Fragment length**  **(bp)** |
| --- | --- | --- | --- |
| rs1922242 | F: AAAGGAAACTGGAGGTATAC  R: CTTGTCAGGTTCTGAGTACC | 52 | 300 |
| rs2235048 | F: TGAGAACATTGCCTATGGAG  R: AAGGAGGGTCAGGTGATCAG | 52 | 302 |
| rs1045642 | F: TCAAAGTGTGCTGGTCCTG  R: ACAAGGAGGGTCAGGTGATC | 63 | 452 |

**Table 1-** **source data 2 Primer sequences used in genotyping analysis for *ABCB1*.**
