# Supplementary material for: Gut microbiota induces high platelet response in patients with ST segment elevation myocardial infarction after ticagrelor treatment
Source: eLife. 2022 Mar 8;11:e70240. doi: 10.7554/eLife.70240 (PMC8903831; doi:10.7554/eLife.70240)
Supplement: Figure 2—source data 1. [file elife-70240-fig2-data1.pdf]

# 16S rDNA Amplicon测序
# 结题报告

客户：童方念&张效林

项目编号：RY20180105A006

上海锐翌生物科技有限公司

2018-02-07

# 一、项目信息

| 项目相关信息 | | | |
|---|---|---|---|
| 项目名称 | 沈阳军区总医院25个人肠道微生物16S rDNA测序分析 | | |
| 项目编号 | RY20180105A006 | | |
| 样品来源 | 人肠道 | | |
| 样品类型 | DNA | | |
| 备注信息 | XXX | | |
| 客户信息 | | | |
| 项目联系人 | 童方念 | 联系电话 | 18840107238 |
| | | 邮箱 | fangnian2369@163.com |
| 单位名称 | 沈阳军区总医院 | | |
| 单位地址 | 沈阳市沈河区文化路83号 | | |
| 锐翌科技代表信息 | | | |
| 科技代表 | 张程翔 | 联系电话 | 18204068862 |
| | | 联系邮箱 | zhangcx@realbio.cn |

锐翌基因 REALGENE

# 二、实验流程

## 2.1 Illumina 测序实验流程

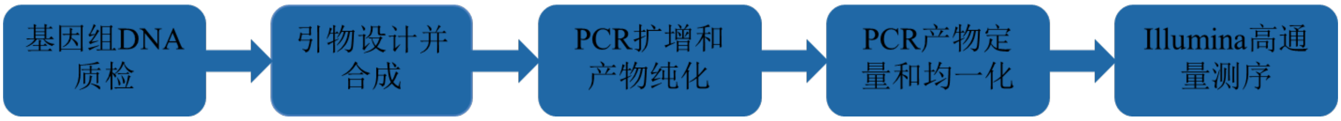


## 2.2 基因组DNA抽提和质检

利用Thermo NanoDrop 2000紫外微量分光光度计和1% 琼脂糖凝胶电泳进行总DNA质检。

## 2.3 引物设计并合成

16S rDNA扩增选择区域为V3-V4区，使用的通用引物为F341和R806。在通用引物的5’端加上适合HiSeq2500 PE250测序的index序列和接头序列，完成特异性引物的设计。

forward primer（5’-3’）：ACTCCTACGGGRSGCAGCAG （F341）

reverse primer（5’-3’）：GGACTACVVGGGTATCTAATC （R806）

## 2.4 PCR扩增和产物纯化

以稀释后的基因组DNA为模板，使用KAPA HiFi Hotstart ReadyMix PCR kit高保真酶进行PCR，确保扩增的准确性和高效性。 用2%琼脂糖凝胶电泳检测PCR产物，并用AxyPrep DNA凝胶回收试剂盒（AXYGEN公司）切胶回收PCR产物。 回收后，利用Thermo NanoDrop 2000紫外微量分光光度计和2%琼脂糖凝胶电泳进行文库质检。

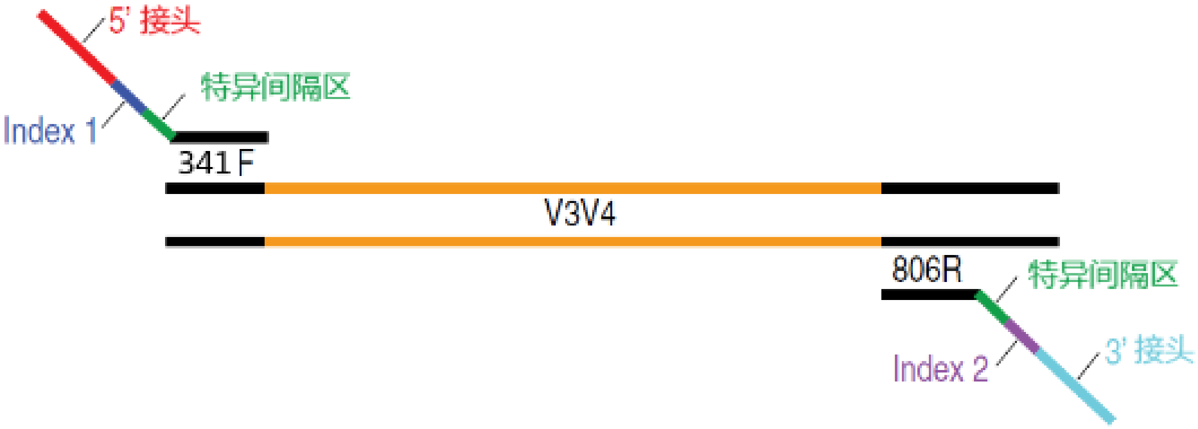


## 2.5 PCR产物定量和均一化

文库质检合格后，使用Qubit进行文库定量，并根据每个样品的数据量要求，进行相应比例的混合。

## 2.6 Illumina 测序

使用Illumina HiSeq PE250进行上机测序。

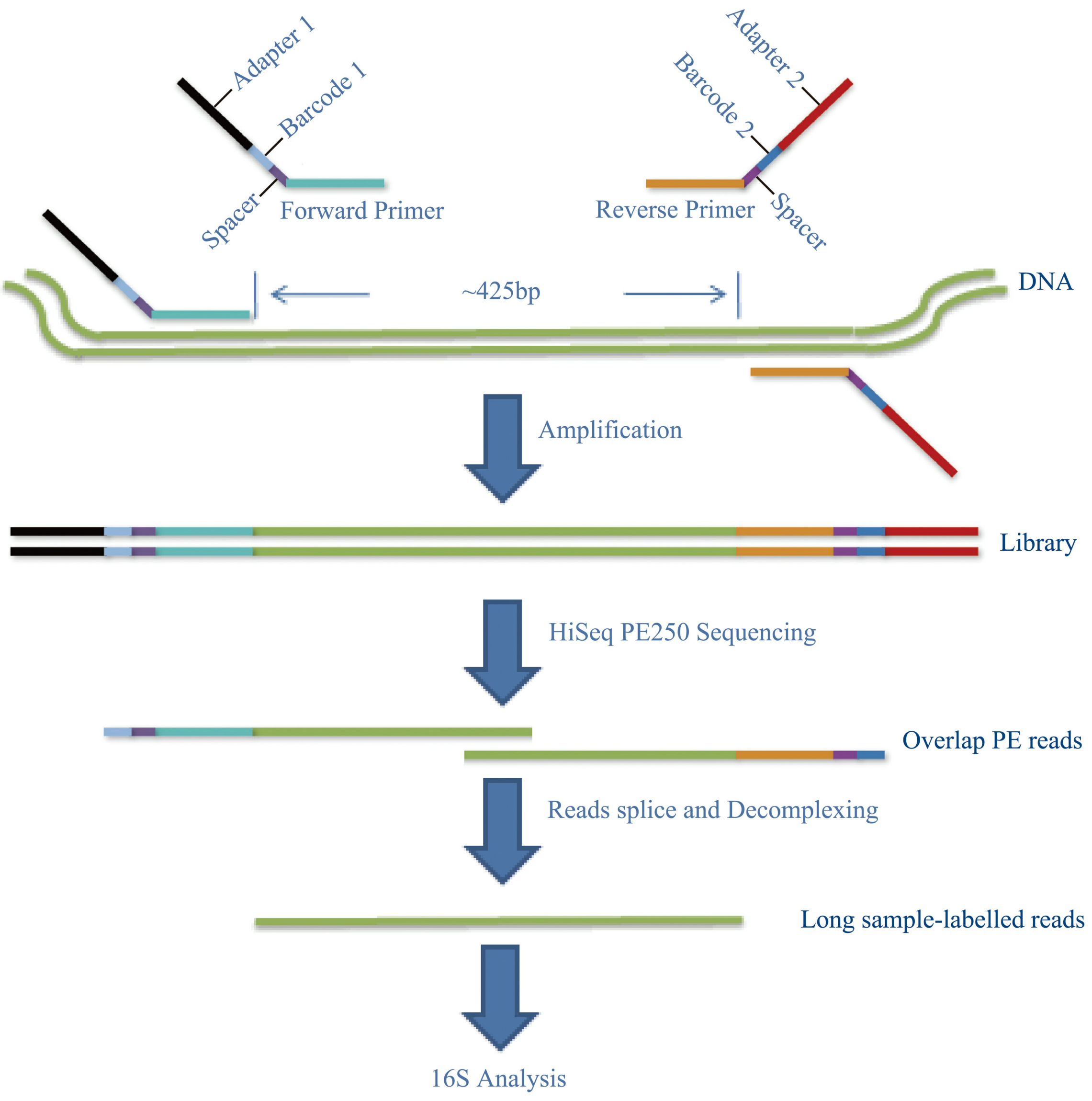


图2-6-1 测序原理图

设计16S特定引物扩增特异区域，得到 425bp 左右扩增片段。加接头，采用HiSeq平台，测序得到2X250bp的Paired-End数据，通过拼接，可以得到较长序列，从而进行16S分析。

# 三、生物信息分析流程

## 3.1生物信息分析流程

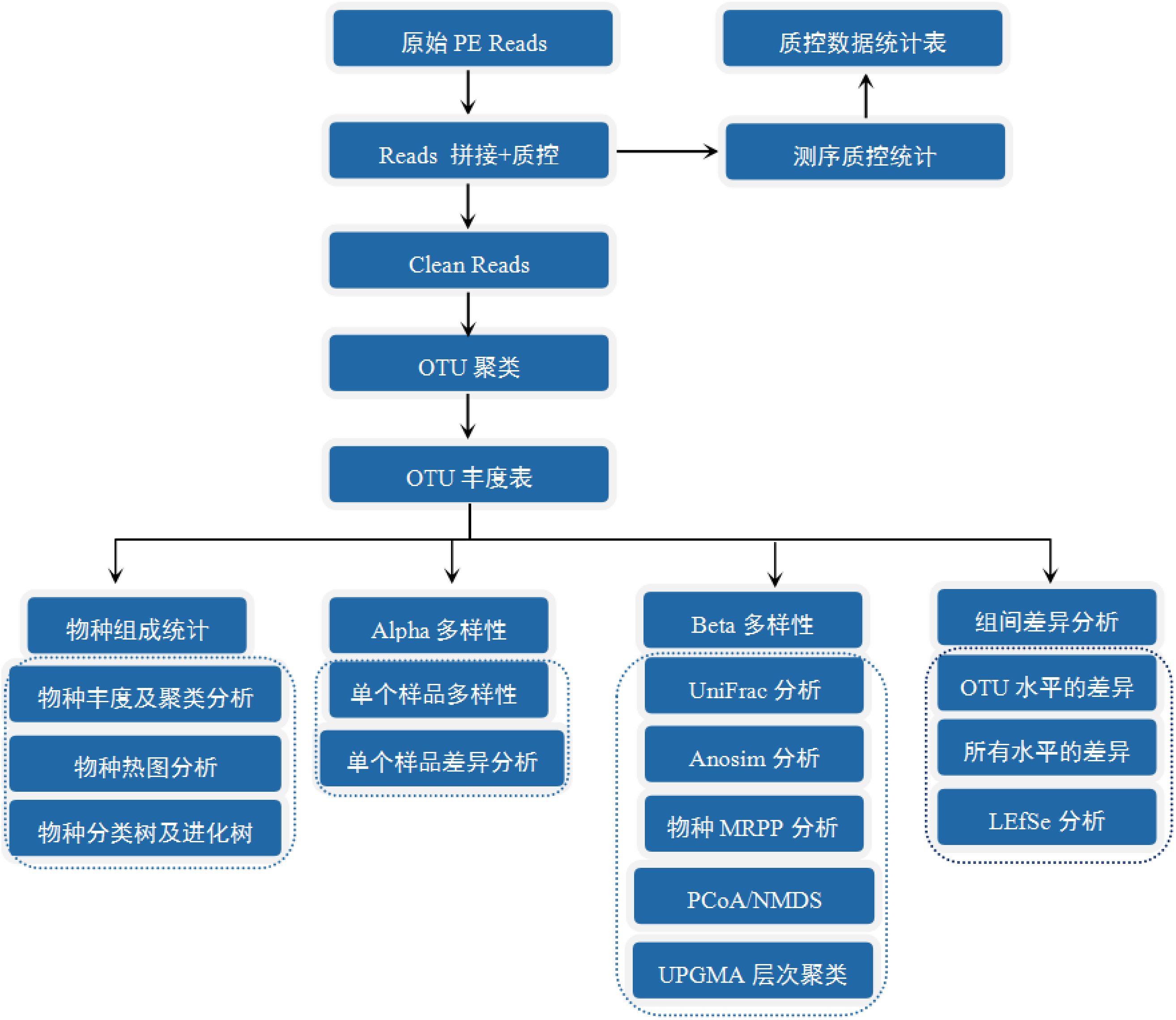


图3-1-1 分析流程图

对原始数据进行QC之后，用Usearch软件对数据进行去嵌合体和聚类的操作，Usearch聚类时，先将Reads按照丰度从大到小排序，通过97%相似度的标准聚类， 得到OTU(Operational Taxonomic Units)，每个OTU被认为可代表一个物种。接下来对每个样品的Reads进行随机抽平处理， 并提取对应的OTU序列。然后使用Qiime软件，做Alpha多样性指数的稀释曲线，根据稀释曲线选择合理的抽平参数，利用Qiime软件对得到的抽平后的OTU进行分析，首先从OTU中分别提取一条Read作为代表序列， 使用RDP方法，将该代表序列与16S数据库比对，从而对每个OTU进行物种分类。归类后，根据每个OTU中序列的条数，从而得到OTU丰度表，最后根据该OTU丰度表进行后续分析。

3.2项目分析内容

| | 一、OTU分析 | | 二、物种分类与丰度分析 | | 三、Alpha分析 |
|---|---|---|---|---|---|
| ☑ | 数据格式说明 | ☑ | 物种注释分析 | ☑ | 单样品多样性分析 |
| ☑ | 有效数据统计 | ☑ | 物种注释结果统计 | ☑ | 单个样品差异分析 |
| ☑ | 数据优化统计 | ☑ | star分析 | | |
| ☑ | OTU聚类 | ☑ | 物种聚类分析 | | |
| ☑ | 抽平处理 | ☑ | 物种热图分析 | | |
| ☑ | Core microbiome分析 | ☑ | Rank Abundance曲线 | | |
| ☑ | OTU Venn 图分析 | ☑ | 物种分类树分析 | | |
| ☑ | OTU PCA分析 | ☑ | Krona分析 | | |
| ☑ | Specaccum物种累积曲线 | ☑ | 物种进化树分析 | | |
| | 四、Beta多样性分析 | | 五、显著差异分析 | | 六、个性化分析 |
| ☑ | UniFrac 热图分析 | ☑ | 物种 LEfSe 差异分析 | ☐ | 物种CCA/RDA分析 |
| ☑ | Anoism分析 | ☑ | 组间秩和检验分析 | ☐ | 物种STAMP差异分析 |
| ☑ | 物种MRPP分析 | | | ☐ | 16S功能预测 |
| ☑ | PCoA分析 | | | ☐ | Network网络分析 |
| ☑ | 非度量多维度分析(NMDS) | | | | |
| ☑ | UPGMA层次聚类 | | | | |

设计到的所以分析内容全部列表，然后“ √ ”选项目设计到的分析内容

锐翌基因 REALGENE

# 四、测序数据统计

## 4.1 数据格式说明

Paired-end 测序，*_1.fq 和*_2.fq 分别对应一个FASTQ 文件。FASTQ 文件是用户得到的最原始文件，其中每4 行表示一条read，即：

@FC4290FAAXX:4:1:3:84#CAGATC/1

CCAACTATGATAGCCAANAAGGGAAAGCCATAGAG…

+

abb_aab_aa`a^aba^D[`a_`aaaa`_a_`__a…

每个序列共有4 行，

第1行是@序列ID，包括index 序列及read1 或read2 标志，由测序仪产生；

第2行是碱基序列，大写“ACTGN”；第三行是“+”，省略了序列ID；

第3行是一个 + 号；

第4行是序列对应的测序质量值序列，每个字母对应第2 行每个碱基，第四行每个字母对应的ASCII 值减去33，即为该碱基的测序质量值；

比如I 对应的ASCII 十进制值为73，那么其对应的碱基质量值是40。Solexa碱基质量值范围为0 到41。

## 4.2 有效数据统计

通过Illumina平台进行Paired-End测序，Paired End Reads通过Reads之间的Overlap关系拼接成长Reads，并对拼接后的Reads进行质控，得到Clean Reads。

软件平台：拼接: Pandaseq[1] ；质控：锐翌分析平台

结果文件： 04_data_statistics/reads_stat.tsv

；

3）Reads长度范围为220~500 nt。

表4-2-1 有效序列统计

| sampleName | cleanReads | Bases | Q20 | Q30 | GC | averageLength |
|---|---|---|---|---|---|---|
| B-26 | 63329 | 26408677 | 95.84% | 93.05% | 50.04% | 417.00 |
| A-24 | 63044 | 26632542 | 95.99% | 93.15% | 52.14% | 422.00 |
| A-25 | 60001 | 25091226 | 96.10% | 93.23% | 55.65% | 418.00 |
| A-26 | 54058 | 22270632 | 96.32% | 93.64% | 51.40% | 411.00 |
| A-20 | 62699 | 26525291 | 95.93% | 93.09% | 51.53% | 423.00 |
| A-21 | 57532 | 24185450 | 96.22% | 93.51% | 50.55% | 420.00 |
| A-22 | 55655 | 23279032 | 96.23% | 93.54% | 50.69% | 418.00 |
| A-23 | 59216 | 24682712 | 95.97% | 93.18% | 51.54% | 416.00 |
| B-14 | 59857 | 24891542 | 94.72% | 91.21% | 50.65% | 415.00 |
| B-2 | 55145 | 23221241 | 94.49% | 90.40% | 49.39% | 421.00 |

注：第一列Sample name 是样品名称

第二列Clean Reads是有效序列数

第三列Bases(bp) 是总碱基数

第四列 Q20 (%) 是Q20碱基占总碱基数的百分比

第五列Q30 (%) 是Q30碱基占总碱基数的百分比

第六列)GC (%) 是该样品的GC含量

第七列Average length(bp) 是指该样品的平均长度

## 4.3 数据优化统计

对16S rDNA高变区测序序列进行测序，测序为V3-V4区；通过Pandaseq软件利用重叠关系将双末端测序得到的成对Reads拼接成一条序列，得到高变区的长Reads。然后使用内部撰写的程序对拼接后的Reads进行如下处理，获取Clean Reads：

1）去除平均质量值低于20的Reads；

2）去除Reads含N的碱基数超过3个的Reads；

3）Reads长度范围为220~500 nt。

软件平台：锐翌分析平台

结果文件： 04_data_statistics/length_distrubution.tsv

表4-3-1 有效序列的长度分布

| length_name | num |
|---|---|
| 220-240 | 0 |
| 240-260 | 443 |
| 260-280 | 119 |
| 280-300 | 60 |
| 300-320 | 108 |
| 320-340 | 69 |
| 340-360 | 53 |
| 360-380 | 88 |
| 380-400 | 4147 |
| 400-420 | 891160 |

第一列length：碱基长度；

第二列sequences：reads数量；

第三列Percent是占总序列的百分比。

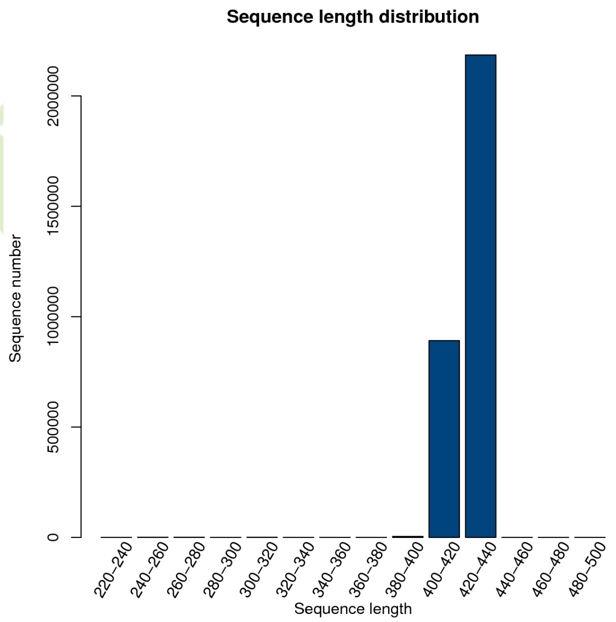


图4-3-1 序列长度的统计

注：横坐标是序列的长度，纵坐标是Reads的数量

# 五、OTU分析

## 5.1 OTU聚类

首先我们将序列完全一样的Clean Reads根据其丰度大小进行排序，将其中的Singletons过滤掉，（因为Singletons可能由于测序错误造成，故将这部分序列去除，不进行后期OTU聚类），利用Usearch在0.97相似度下进行聚类，对聚类后的序列进行嵌合体过滤后，得到用于物种分类的OTU(Operational Taxonomic Units)，最后将所有Clean Reads比对到OTU序列上，将能比对上OTU的Reads提取出来，得到最终的Mapped Reads[2]-[3]。统计各个样品每个OTU中的丰度信息，OTU的丰度初步说明了样品的物种丰富程度。

52个样品共产生975个OTU。

软件平台：usearch

结果文件： pick_otu_summary.tsv

表5-1-1 样品数据和OTU统计

| sampleName | ampliconType | cleanReads | mappedReads | mappedRatio | OTUs |
|---|---|---|---|---|---|
| A-8 | 16S | 61225 | 52391 | 85.57% | 235 |
| A-7 | 16S | 64294 | 56151 | 87.33% | 146 |
| A-6 | 16S | 60902 | 48234 | 79.20% | 209 |
| A-5 | 16S | 50668 | 45306 | 89.42% | 231 |
| A-4 | 16S | 49724 | 42569 | 85.61% | 239 |
| A-3 | 16S | 57255 | 50132 | 87.56% | 146 |
| A-26 | 16S | 54058 | 38263 | 70.78% | 295 |
| A-25 | 16S | 60001 | 51779 | 86.30% | 186 |
| A-24 | 16S | 63044 | 56444 | 89.53% | 154 |
| A-23 | 16S | 59216 | 48982 | 82.72% | 225 |

注：第一列Sample Name是样品名称；

第二列Amplicon type是扩增子类型；

第三列Clean Read是质控后的Reads条数(合同中的clean tags)；

第四列Mapped Reads是比对上OTU的Reads占Clean Reads的数量；

第五列Mapped Reads(%)是比对上OTU的Reads占Clean Reads的比例；

第六列OTUs是样品所含有的OTU个数。

## 5.2 抽平处理

不同样本对应的Reads数量差距较大，为避免因样品数据大小不同而造成分析时的偏差，我们在样品达到足够的测序深度的情况下，对每个样品进行随机抽平处理。测序深度用Alpha多样性指数来衡量。抽平的参数必须在保证测序深度足够的前提下去选取。

observed_species指数表示实际观测到的OTU数量.

chao1指数用来估计样品所含OTU的总数，其公式为：

$$S_{chao1} = S_{obs} + \frac{n_1(n_1-1)}{2(n_2+1)}$$

Schao1：估计的OTU数量；

Sobs：实际观察到的OTU数量；

n1：只含一条序列的OTU的数量；

n2：只含两条序列的OTU的数量；

参考网址：http://scikit-bio.org/docs/latest/generated/generated/skbio.diversity.alpha.chao1.html

软件平台：锐翌分析平台

结果文件：05_OTU_analysis/otu_downsize_stat.tsv

根据Alpha多样性分析，兼顾测序饱和度和样品完整性，我们对每个样品随机抽取36457条reads。抽平后的样品OTU统计见OTU_downsize_stat.tsv中的样品抽平OTU统计表单，结果如下：

表5-2-1 抽平后样品OTU统计

| sampleName | downsize | otus_before | otus_after |
|---|---|---|---|
| B-9 | 36457 | 151 | 139 |
| B-8 | 36457 | 233 | 210 |
| B-7 | 36457 | 323 | 305 |
| B-6 | 36457 | 357 | 333 |
| B-5 | 36457 | 238 | 238 |
| B-4 | 36457 | 273 | 253 |
| B-3 | 36457 | 187 | 180 |
| B-26 | 36457 | 246 | 222 |
| B-25 | 36457 | 246 | 230 |
| B-24 | 36457 | 277 | 264 |

注：第一列Sample Name是样品名称；

第二列Downsize是抽平数量；

第三列otu_berfore抽平前的OTU个数；

第四列otus_after是抽平后样品所含有的OTU个数。

Alpha多样性反映的是单个样品内部的物种多样性，包括observed species指数和chao指数。

结果目录：04_data_statistics\alpha

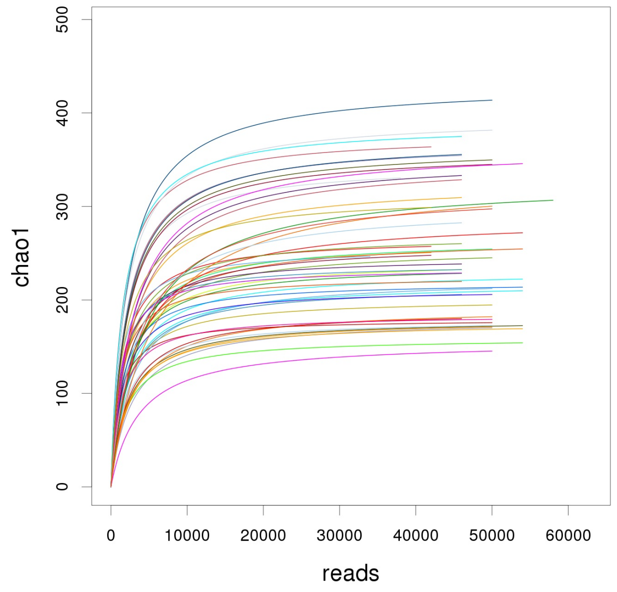


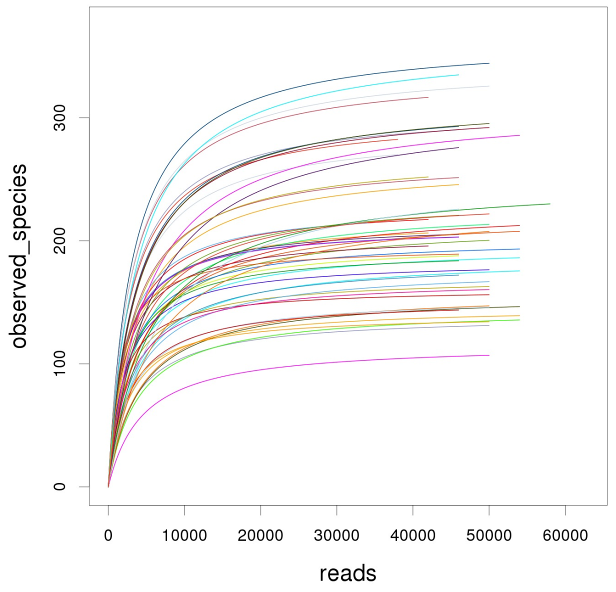


图5-2-1 表示样品物种丰富度Alpha多样性指数稀释曲线图

注：横轴表示从某个样品中随机抽取的Clean Reads数目，纵轴表示该Reads数目对应的Alpha多样性指数的大小。图中一条曲线代表一个样品，随着测序深度的增加，当曲线趋于平缓时表示此时的测序数据量比较合理。

## 5.3 Core microbiome分析（总样品数≥5）

根据样品的共有OTU以及OTU所代表的物种，可以找到Core microbiome（覆盖100%样品的微生物组）。

软件平台：锐翌分析平台

结果文件： 05_OTU_analysis\core_otu\core_otu.tsv

表5-3-1 Core microbiome OTU列表

| otuId | taxonomyLevel | taxonomyName |
|---|---|---|
| denovo72 | genus | Alistipes |
| denovo35 | genus | Parabacteroides |
| denovo89 | genus | Blautia |
| denovo1 | genus | Bacteroides |
| denovo3 | genus | Escherichia/Shigella |
| denovo4 | genus | Bacteroides |
| denovo703 | genus | Bacteroides |
| denovo18 | genus | Bacteroides |
| denovo13 | genus | Faecalibacterium |
| denovo17 | genus | Bacteroides |

注：

第一列OTU ID是OTU名称；

第二列Taxonomy Level是OTU所代表的物种所在的分类水平；

第三列Taxonomy Name是OTU所代表的物种名称。

共有OTU数与样品的关系

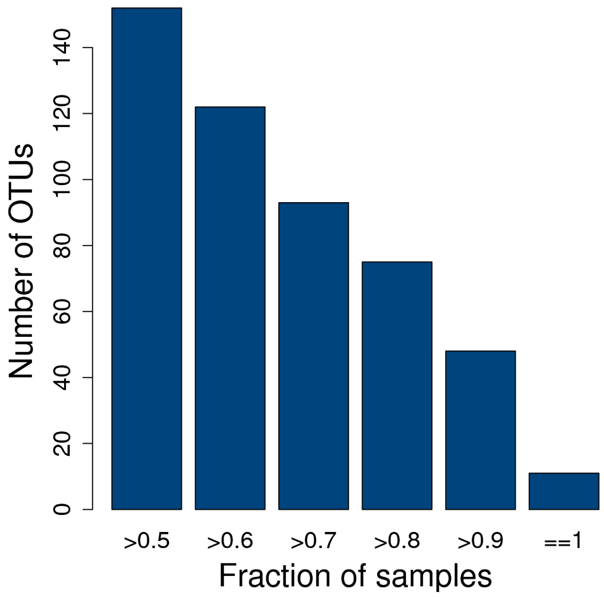


图5-3-1 共有OTU数与样品的关系

注：横坐标是覆盖样品的比例，纵坐标是统计的覆盖大于此比例样品的OTU的数目。图中表示的是覆盖一定比例以上样品的OTU的数目。

## 5.4 OTU flower图分析(分组数量6-10组)

根据每个样品的OTU在每个样品的丰度，计算出每个样品或组间共有和特有的OTU，Venn图可以很好的反应组间共有以及组内特有的OTU数目。

软件平台：R语言/Perl SVG

结果目录： 05_OTU_analysis\venn\

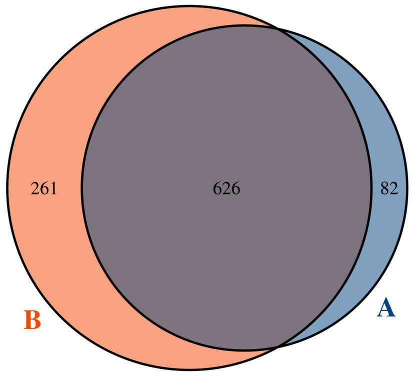


图5-4-1 OTU venn图（2-5组）

注：不同颜色图形代表不同样品或者不同组别，不同颜色图形之间交叠部分数字为两个样品或两个组别之间共有的OTU个数。同理，多个颜色图形之间交叠部分数字为多个样品或组别之间共有OTU个数；Venn图，分组数量：2-5个组；花瓣图分组数量6-10个组。

## 5.5 OTU PCA分析（总样品数≥5）

PCA分析(Principal Component Analysis)，即主成分分析，是一种分析和简化数据集的技术。PCA可以初步的反映出不同处理或不同环境间的样品可能表现出分散和聚集的分布情况，从而可以判断相同条件的样品组成是否具有相似性[5]。

软件平台：R语言

结果目录：05_OTU_analysis\otu_pca\

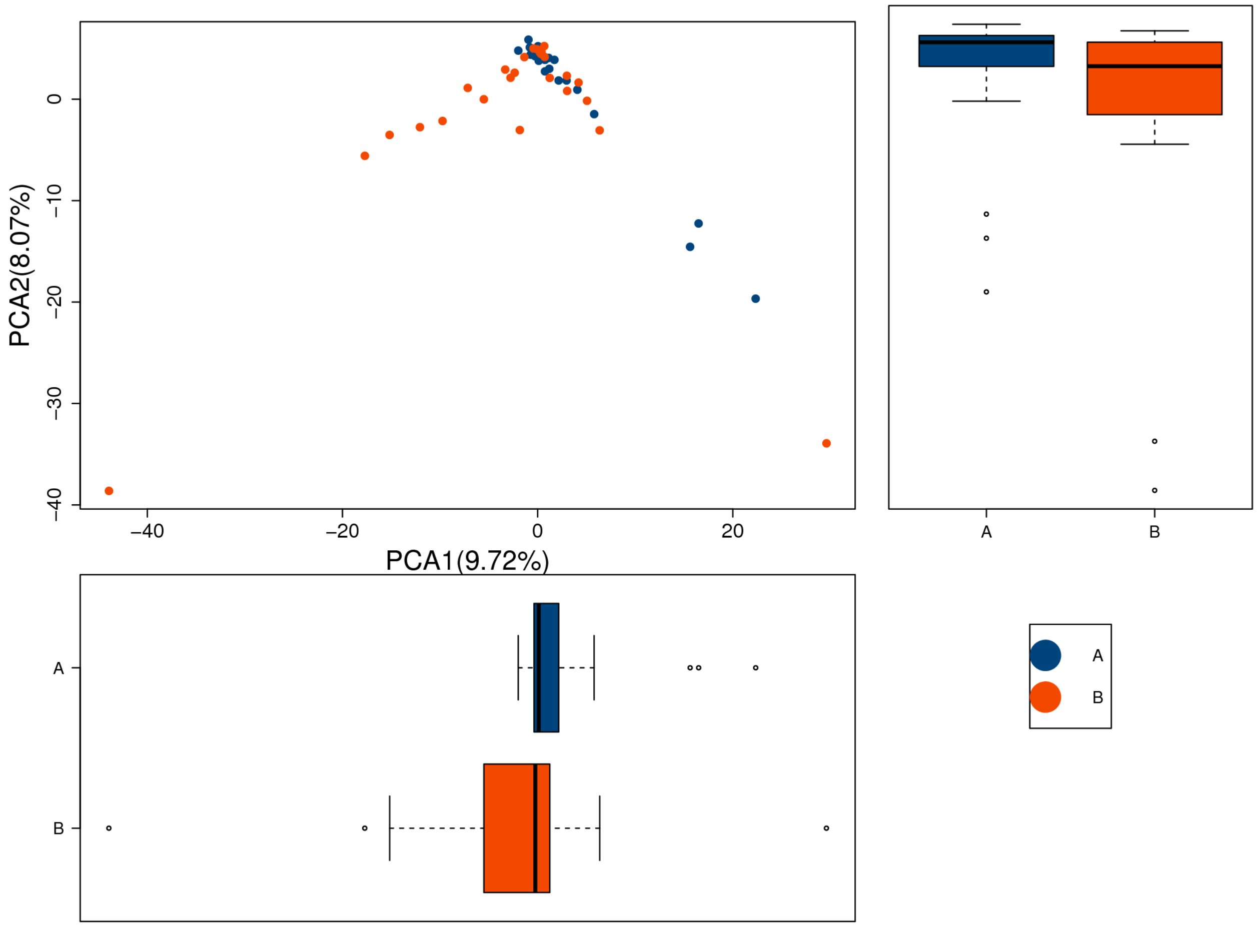


图5-5-1 基于OTU丰度的PCA分析

注：横坐标表示第一主成分，括号中的百分比则表示第一主成分对样品差异的贡献率；

纵坐标表示第二主成分，括号中的百分比表示第二主成分对样品差异的贡献率。

不同颜色代表样品属于不同的分组，两点之间的距离越远，表示两个样品的微生物群落差异越大。

## 5.6 Specaccum物种累积曲线（总样品数≥10）

物种累积曲线(Species Accumulation Curves)用于描述随着抽样量的加大物种增加的状况，是理解调查样地物种组成和预测物种丰富度的有效工具。在生物多样性和群落调查中，被广泛用于抽样量充分性的判断以及物种丰富度(Species Richness)的估计。

利用物种累积曲线判断抽样量是否充分是根据曲线的特征来判断：如果曲线一直急剧上升，几乎为直线，表明抽样量不足，需要增加抽样量；如果曲线在急剧上升后变为上升舒缓，则表明抽样充分。

软件平台：R语言specaccum包

结果目录：05_OTU_analysis\all\specaccum\

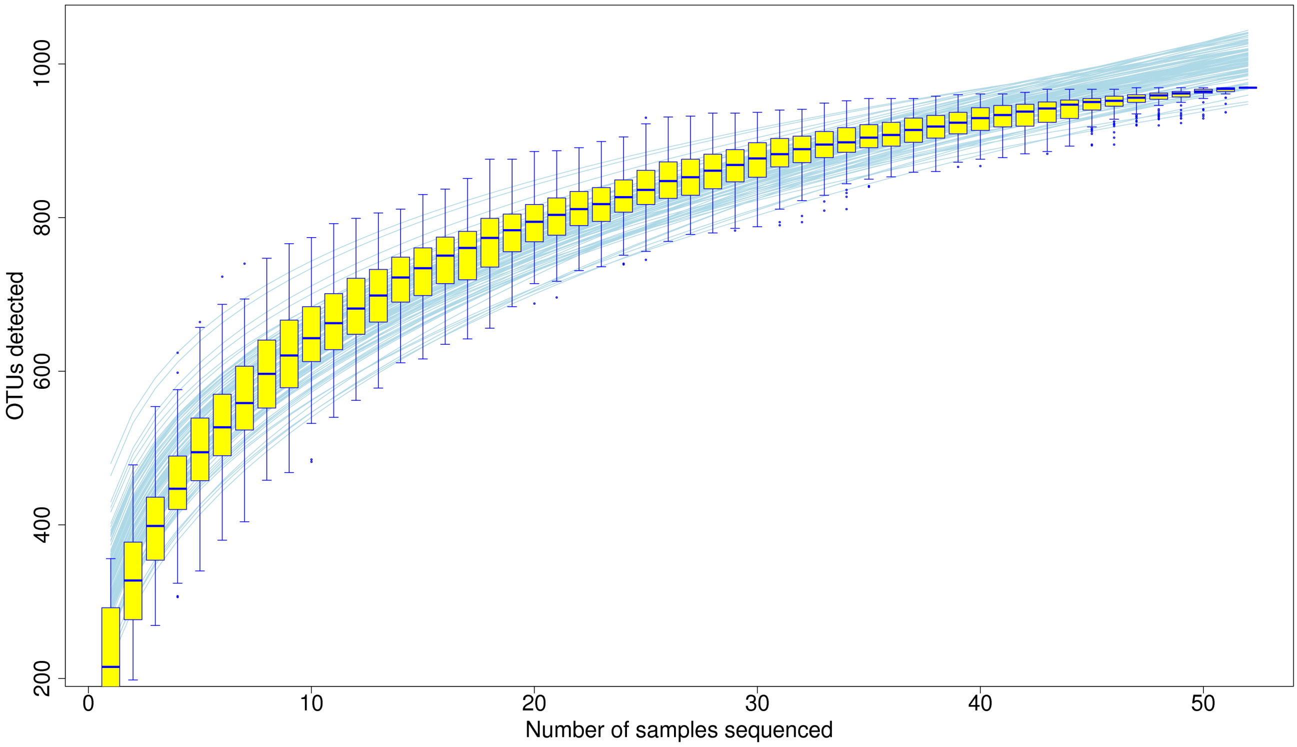


图5-6-1 物种累积曲线

注：横坐标是样品的数量，纵坐标是OTUs的数量。由物种累积曲线可以判断抽样量是否充分，在抽样量充分的前提下，运用物种累积曲线还可以对物种丰富度进行预测。

# 六、物种分类和丰度分析

## 6.1 物种注释分析

从各个OTU中挑选出丰度最高的一条序列，作为该OTU的代表序列（代表序列见文件rep_set.fna）。使用RDP方法，将该代表序列与已知物种的16S数据库进行比对，从而对每个OTU进行物种归类（物种注释结果见文件tax_assignments.tsv）。

表6-1-1 OTU注释统计

| otuName | num |
|---|---|
| Assigned to Kingdom | 969.00 |
| Assigned to Phylum | 940.00 |
| Assigned to Class | 890.00 |
| Assigned to Order | 872.00 |
| Assigned to Family | 782.00 |
| Assigned to Genus | 542.00 |
| Assigned to Species | 0 |
| Min no. of OTUs per sample | 105.00 |
| Max no. of OTUs per sample | 356.00 |
| Mean no. of OTUs per sample | 220.04 |

注：第一列注释到的OTUs名称；

第二列注释的OTUs数量；

## 6.2 物种注释结果统计

根据物种注释结果，分别在门、纲、目、科、属分类等级对各个样品做物种profiling相应的柱状图。形成物种相对丰度柱状图，可以直观地查看各样品在不同的分类等级上相对丰度较高的物种和比例[8]-[11]。

软件平台：锐翌分析平台

结果文件：06_classification_abundance_analysis\bar_plot_sample\

## 6.3 物种丰度分析

根据物种注释结果，分别在门、纲、目、科、属分类等级对各个样品做物种profiling相应的柱状图。形成物种相对丰度柱状图，可以直观地查看各样品在不同的分类等级上相对丰度较高的物种和比例[8]-[11]。

软件平台：锐翌分析平台

结果目录： 06_classification_abundance_analysis\bar_plot_sample\

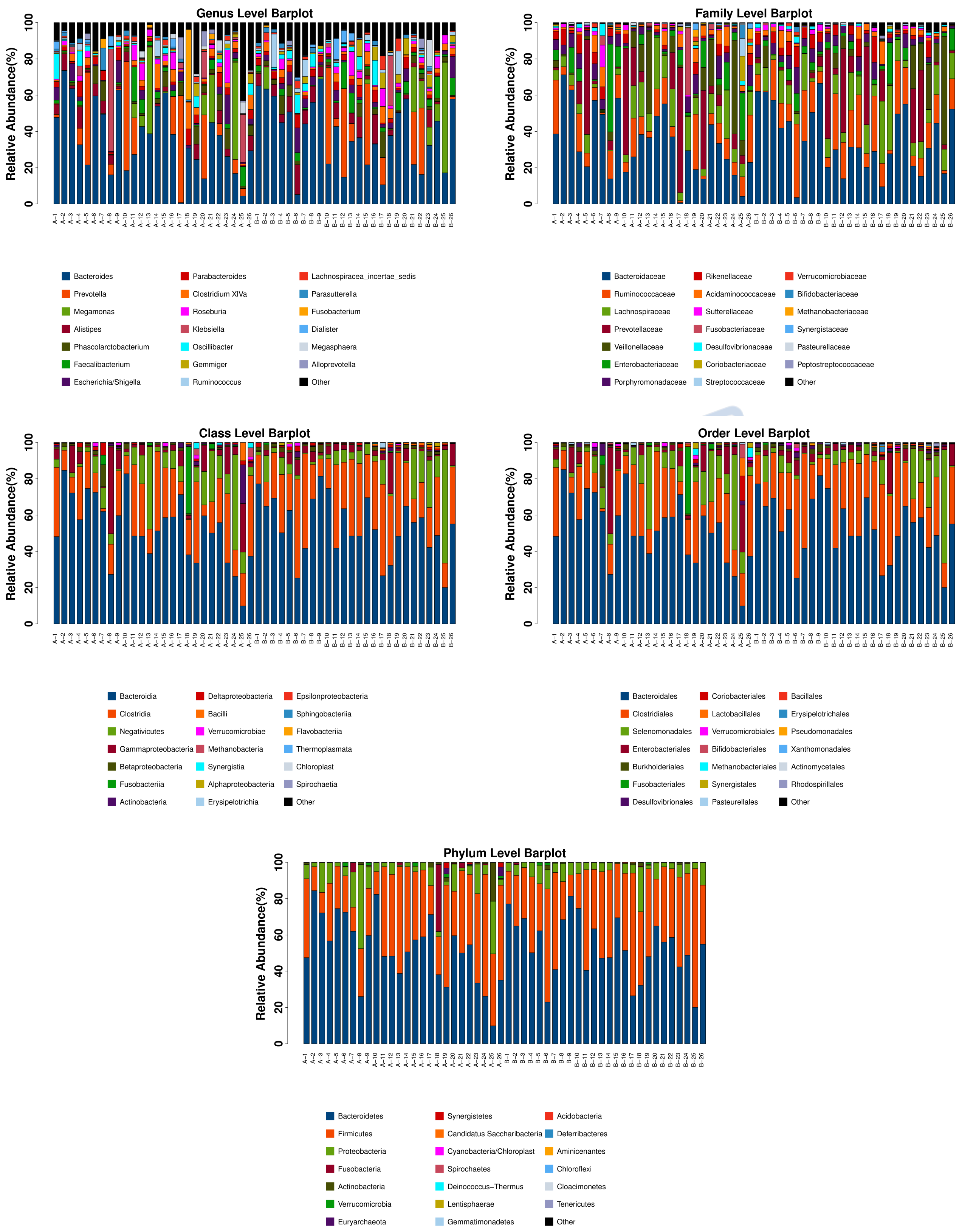


图6-3-1 样品各分类水平中物种Profiling柱状图

注：横轴为样品名称，纵轴为相对丰度的比例。颜色对应不同物种名称，色块长度表示该色块所代表的物种的相对丰度的比例。

根据样品分组信息，分别在门、纲、目、科、属分类等级对不同group做物种profiling相应的柱状图。

软件平台：锐翌分析平台

结果目录： 06_classification_abundance_analysis\bar_plot\

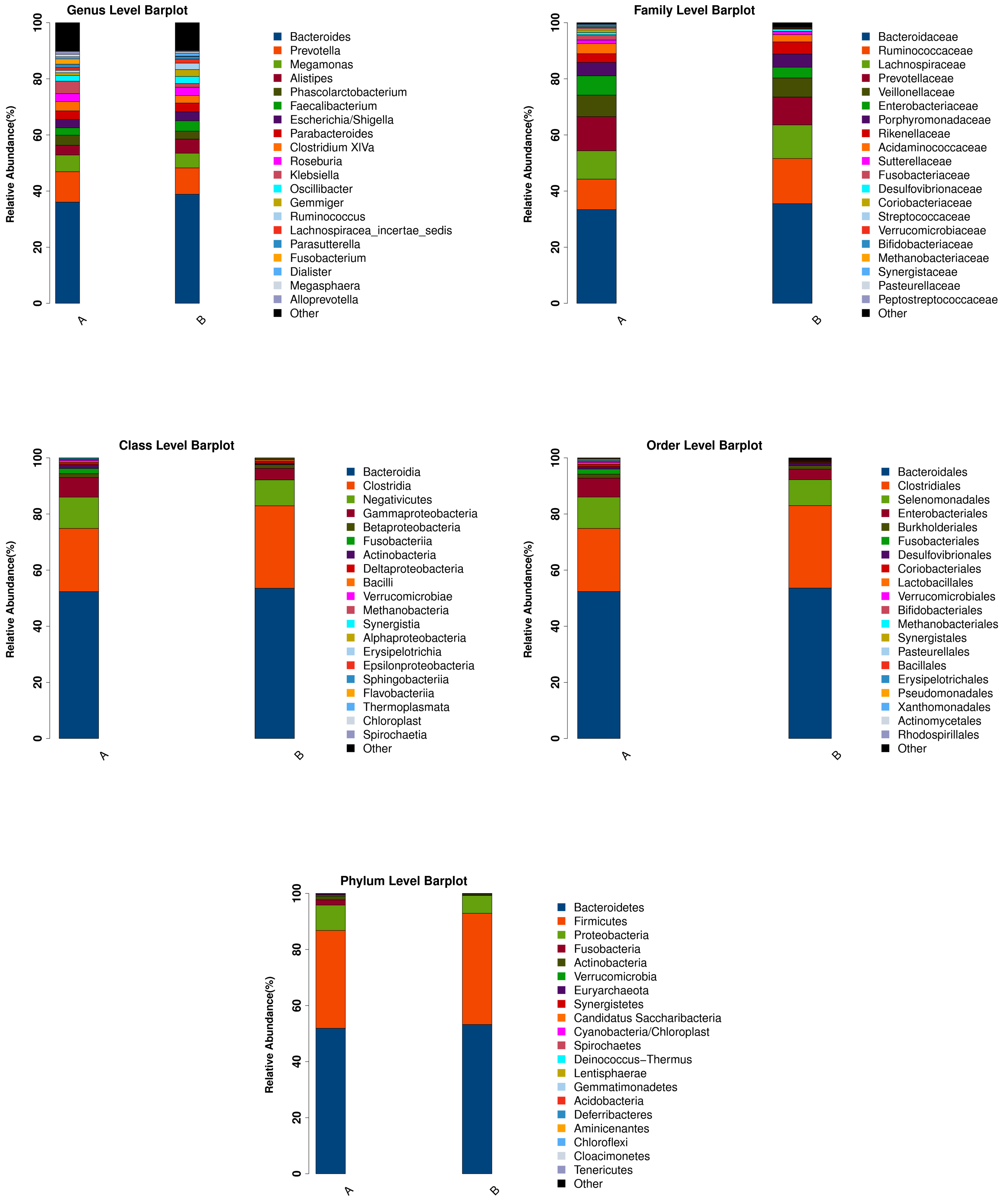


图6-3-2 门分类水平中不同group的物种Profiling柱状图

注：横轴为分组名称，纵轴为相对丰度的比例。颜色对应不同物种名称，色块长度表示该色块所代表的物种的相对丰度的比例。

在属分类等级对10的物种丰度做Star图。一个星形图代表着一个样品的物种相对丰度信息。每个星形图中的扇形代表一个物种，用不同颜色区分，用扇形的半径来代表物种相对丰度的大小，扇形的半径越长表示丰度高，反之丰度相对较低。

软件平台：锐翌分析平台

结果目录： 06_classification_abundance_analysis\tax_star\

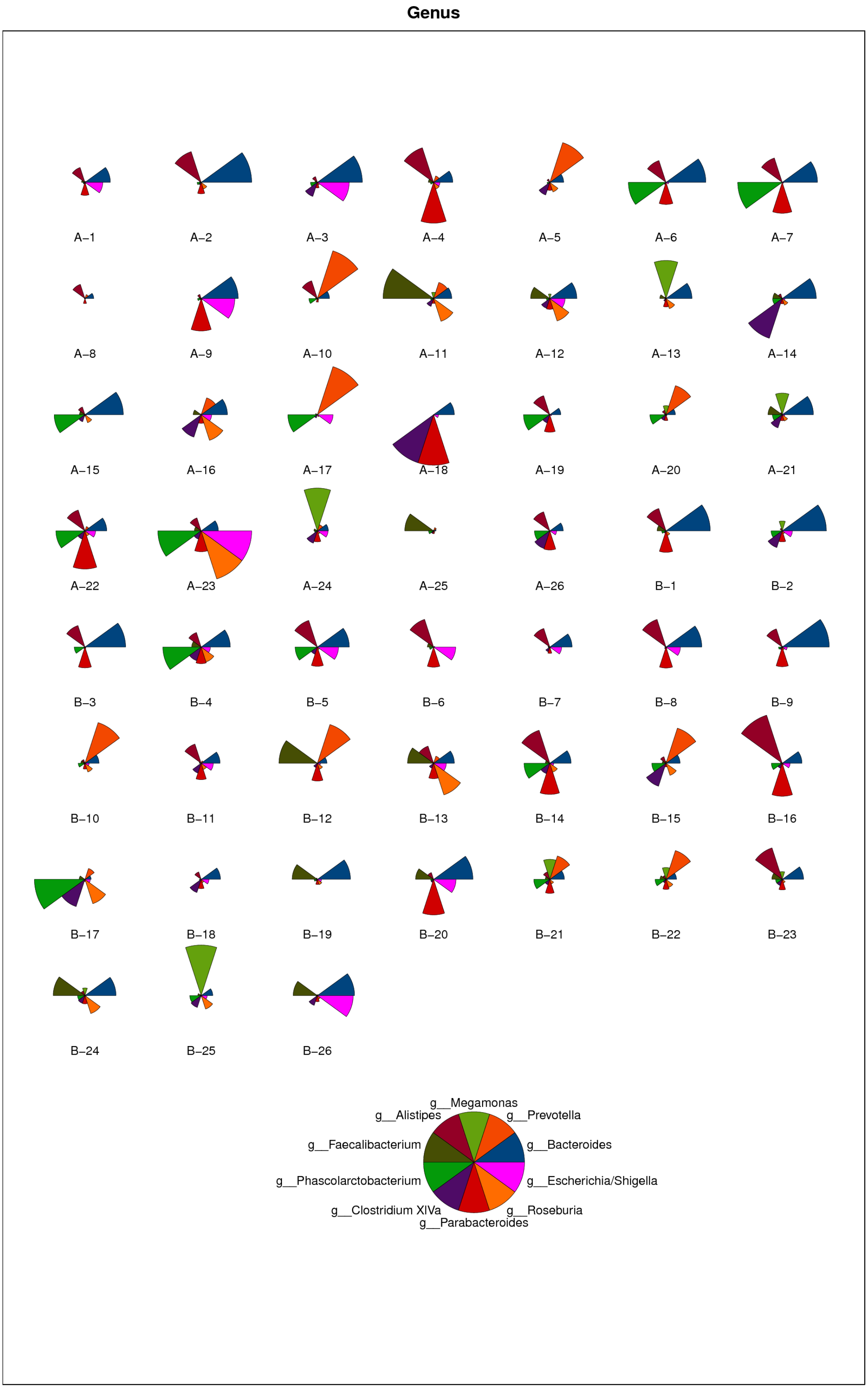


图6-3-6 属分类水平中不同group的top10物种Profiling星图

注： 一个星形图代表着一个样品的物种相对丰度信息。每个星形图中的扇形代表一个物种，用不同颜色区分，用扇形的半径来代表物种相对丰度的大小，扇形半径越长代表此扇形所对应的物种的相对丰度越高。

## 6.4 物种聚类分析

根据样品中物种注释信息及丰度，从物种和样品两个层面进行物种丰度聚类，通过柱状图对比，利于观察样品之间的聚类关系以及物种组成差异。

软件平台：锐翌分析平台

结果目录： 06_classification_abundance_analysis\tax_bar_tree\

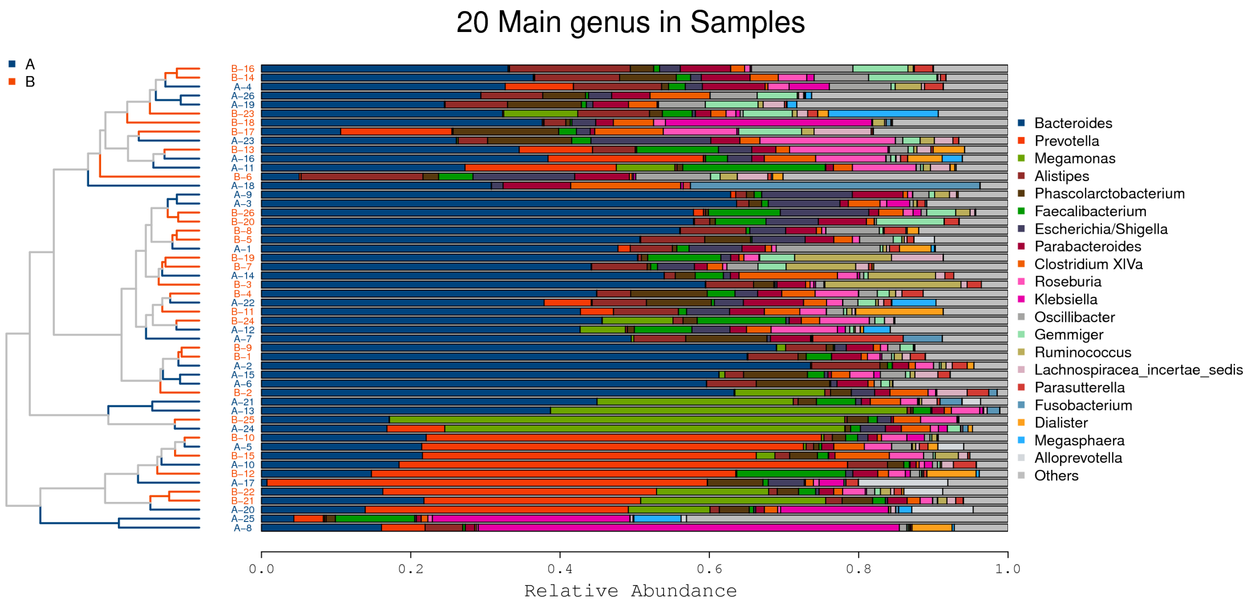


图6-4-1 物种丰度聚类图

注：最右侧为样品聚类结果，颜色区分样品分组；中间为各个样品的所含属水平比例；右侧表示各个颜色代表的属；

## 6.5 物种热图分析（总样品数≥5）

Heatmap是以颜色梯度来代表数据矩阵中数值的大小，并能根据物种或样品丰度相似性进行聚类的一种图形展示方式。 聚类结果加上样品的处理或取样环境分组信息，可以直观地观察到相同处理或相似环境样品的聚类情况，并直接反映了样品的群落组成的相似性和差异性。 本分析内容分别在门，纲，目，科，属分类水平进行Heatmap聚类分析。 纵向聚类表示所有物种在不同样品间表达的相似情况，距离越近，枝长越短，说明样品的物种组成及丰度越相似。横向聚类表示该物种在各样品丰度相似情况， 与纵向聚类一样，距离越近，枝长越短，说明两物种在各样品间的组成越相似[12]-[14]。

软件平台：锐翌分析平台

结果目录： 06_classification_abundance_analysis\heatmap\

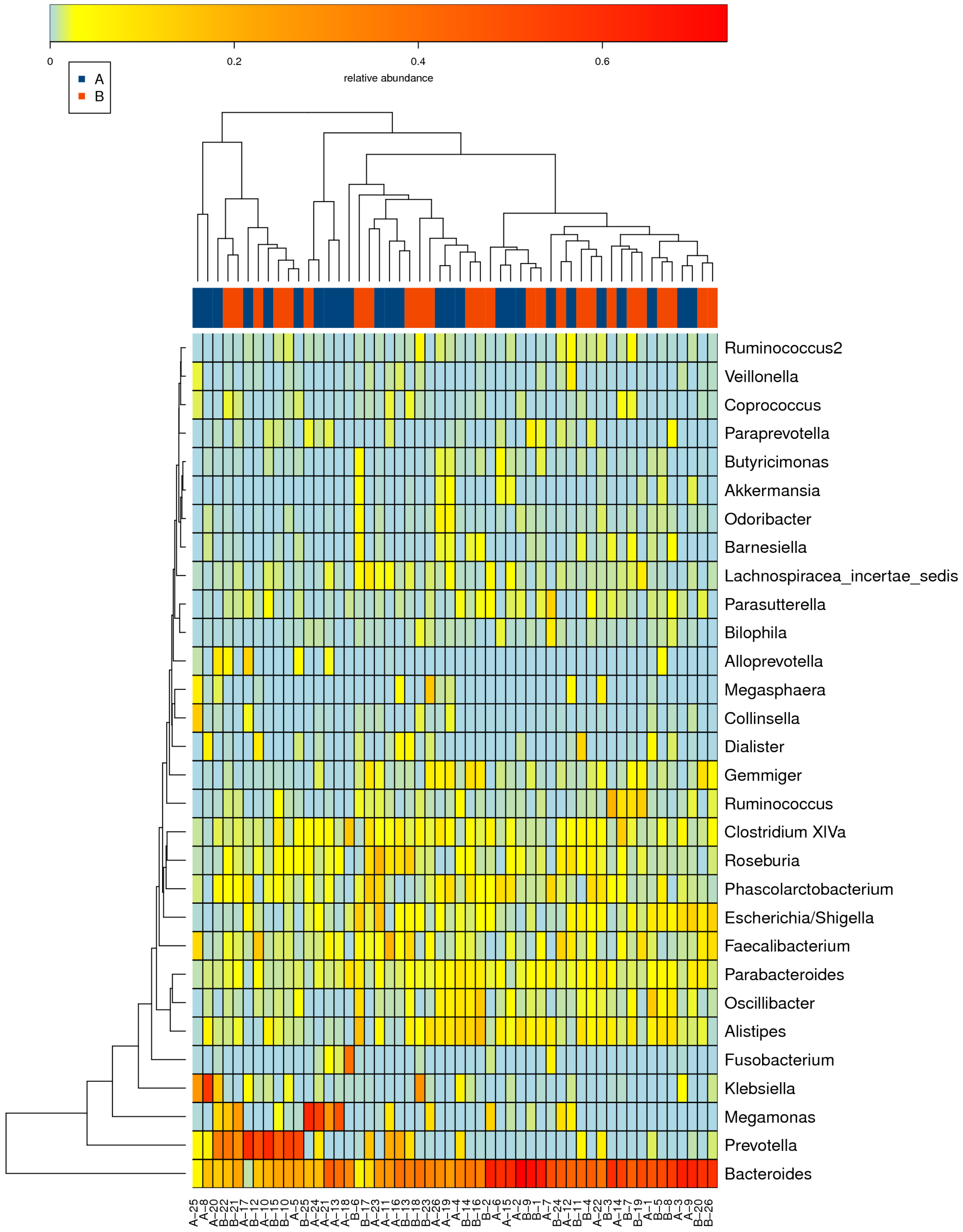


图6-5-1 物种丰度热图

注：横向聚类表示该物种在各样品的丰度相似情况，距离越近，枝长越短，说明两物种在各样品间的组成越相似。纵向聚类表示所有物种在不同样品间表达的相似情况，与横向聚类一样，距离越近，枝长越短，说明两样品的物种组成及丰度越相似。样品分组信息，图中前一行为样品分组信息，颜色与图列对应。

## 6.6 Rank Abundance曲线

将样品中的 OTUs 按相对丰度(或者包含的序列数目)由大到小排序得到对应的排序编号，再以 OTUs 的排序编号为横坐标，OTUs 中的相对丰度(也可用该等级 OTUs 中序列数的相对百分含量)为纵坐标，将这些点用折线连接，即绘制得到Rank

Abundance曲线。

Rank Abundance曲线可同时用于解释样品多样性的物种的丰度和均匀程度两个方面。物种的丰度由曲线在横轴上的长度来反映，曲线越宽，表示物种的组成越丰富；物种组成的均匀程度由曲线的形状来反映，曲线越平坦，表示物种组成的均匀程度越高。

软件平台：qiime

结果目录： 06_classification_abundance_analysis\rank_abundance\

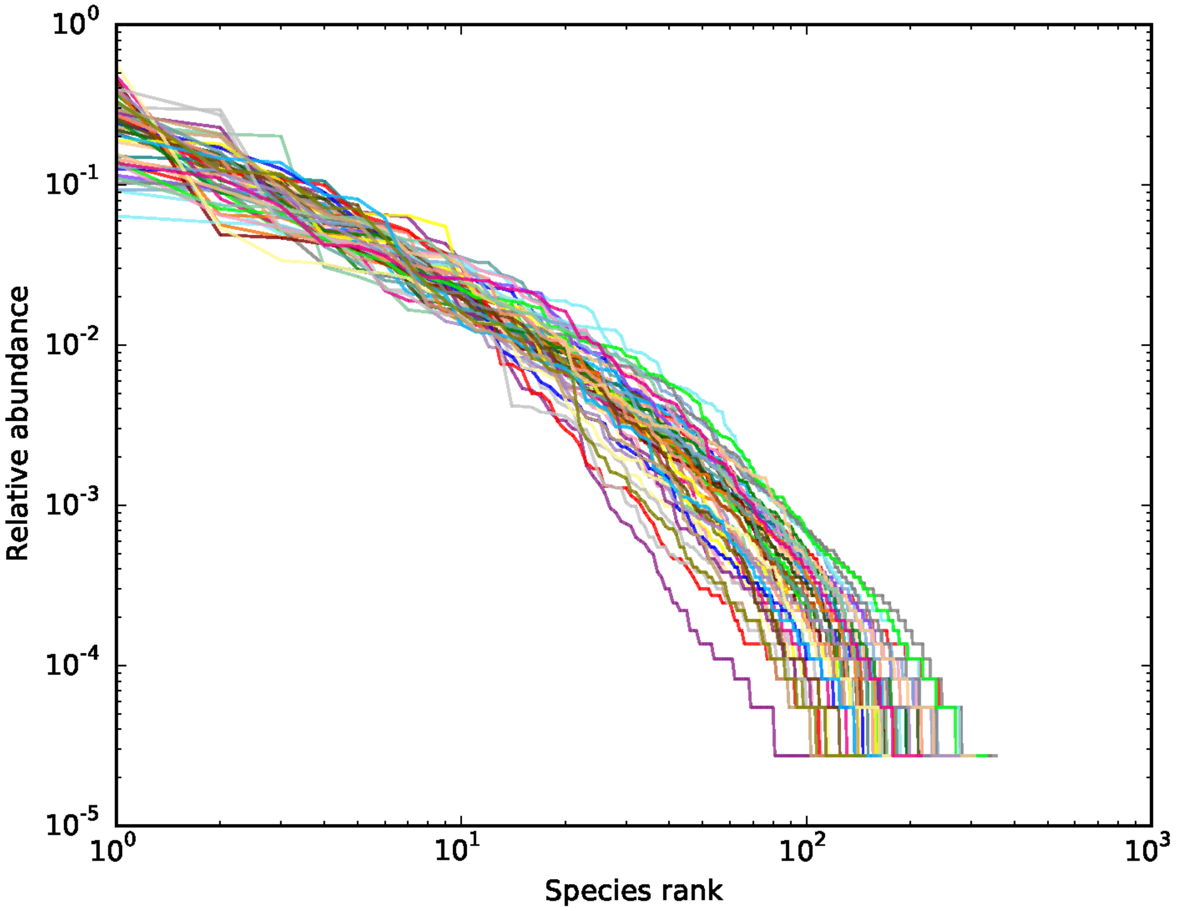


图6-6-1 Rank Abundance曲线

注：横坐标为按 OTUs 丰度排序的序号 ( Rank )，纵坐标为对应的OTUs的丰度 ( Abundance ) ，不同的样品使用不同的颜色和线型的曲线表示。Rank Abundance 曲线在水平方向，分类的丰度由曲线的宽度来反映，分类的丰度越高，曲线在横轴上的跨度越大；在垂直方向曲线的平滑程度，反映了样品中分类的均匀程度，曲线越平缓，物种分布越均匀。

## 6.7 物种分类树

根据物种分类结果，筛选出优势物种，如物种丰度前20进行物种分类树统计，从整个分类系统上了解单个或多个样品中的优势物种的丰度差异和进化关系。

软件平台：锐翌分析平台

结果目录： 06_classification_abundance_analysis\tax_tree\

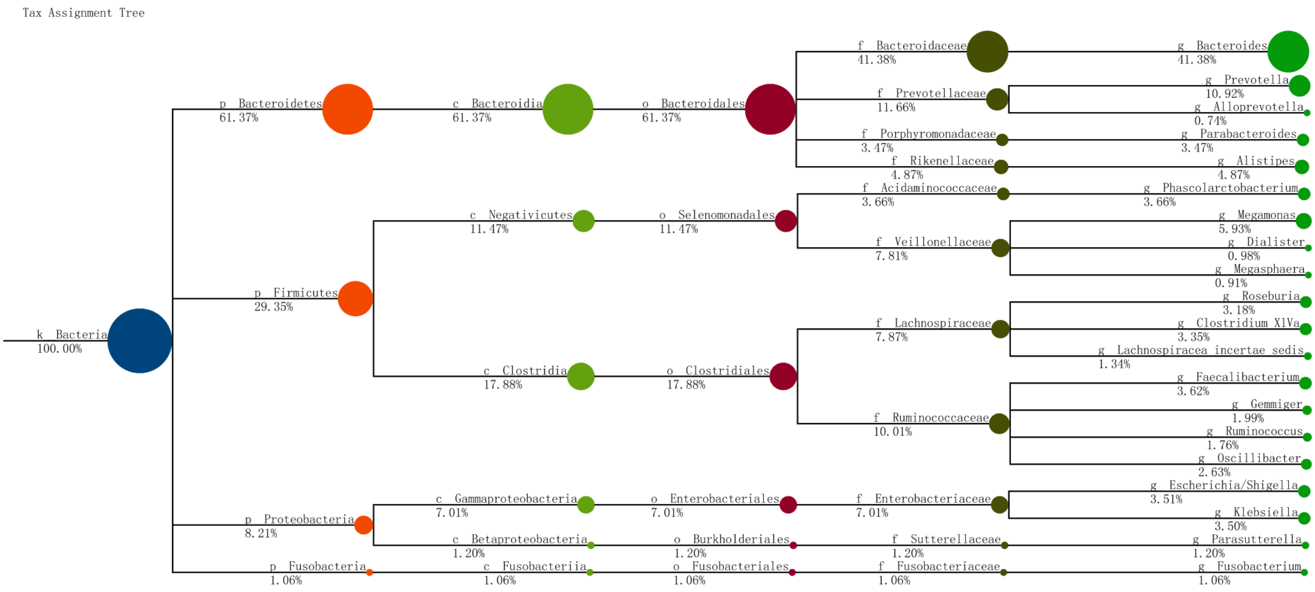


图6-7-1 物种分类以及丰度比例关系图

注：不同颜色表示不同的分类水平，圆圈的大小代表该分类的相对丰度大小；分类名下方的数字表示相对丰度百分率。

每个样品的物种分类树见目录： 样品物种分类树

锐翌基因 REALGENE

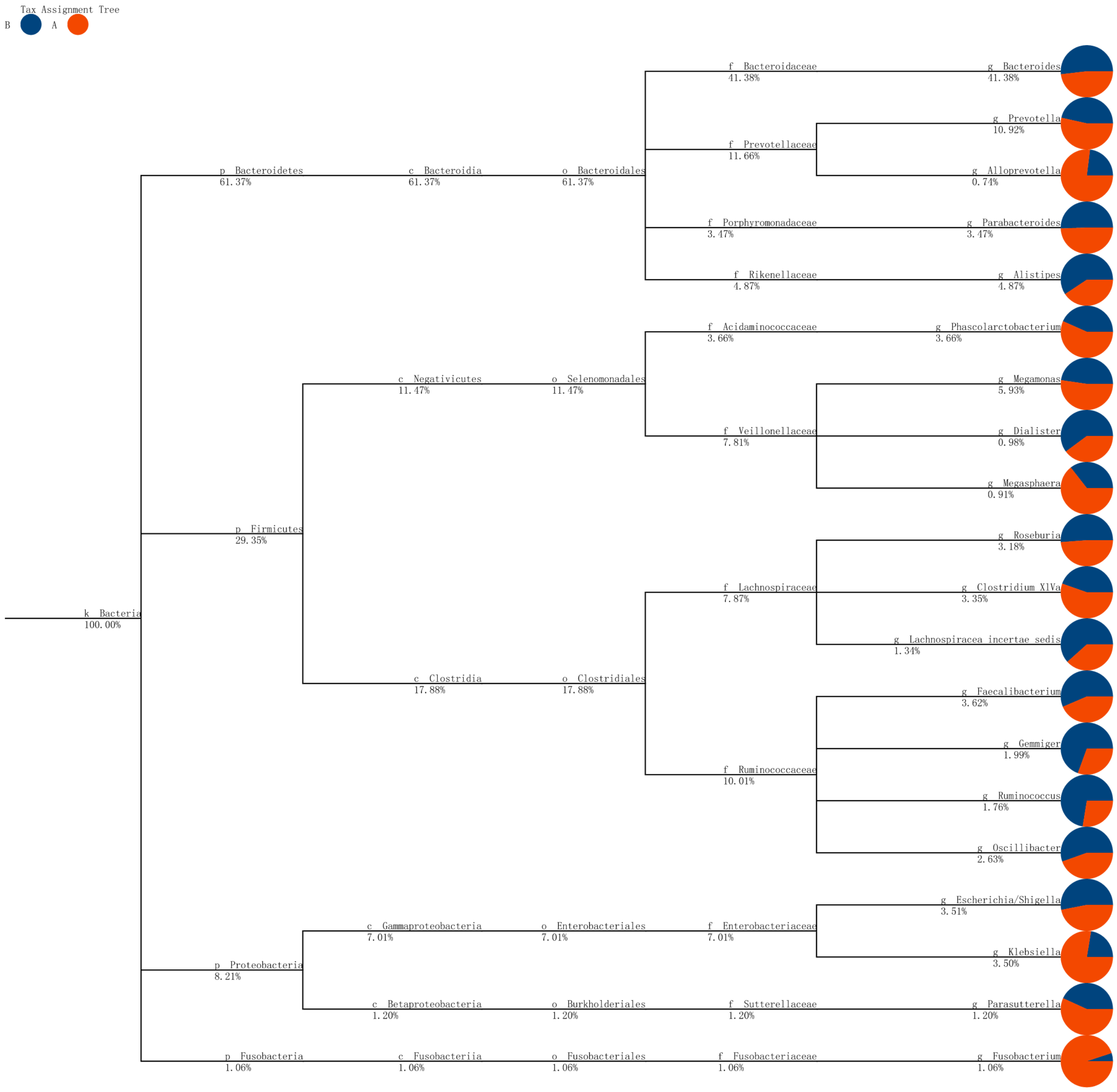


图6-7-2 物种分类以及不同分组占比关系图

注：不同颜色的扇形表示不同的样品/分组，扇形的大小表示该样品/分组在该分类上相对丰度的比例大小；分类名下方的数字表示相对丰度百分率。

## 6.8 物种注释结果KRONA展示

使用KRONA软件对物种注释结果进行可视化展示， 展示结果中，圆圈从内到外依次代表不同的分类级别，扇形的大小代表不同OTU注释结果的相对比例。

软件平台：krona

结果目录： 06_classification_abundance_analysis\krona.html

## 6.9 物种进化树

在分子进化研究中进一步研究系统进化关系，通过系统发生关系推断来揭示某一分类水平OTUs 序列间碱基的差异，结合各个OTUs 序列所代表的物种注释信息，进而构建物种进化树[15]。如下图所示：

软件平台：锐翌分析平台

结果目录： 06_classification_abundance_analysis\phylo_tree\

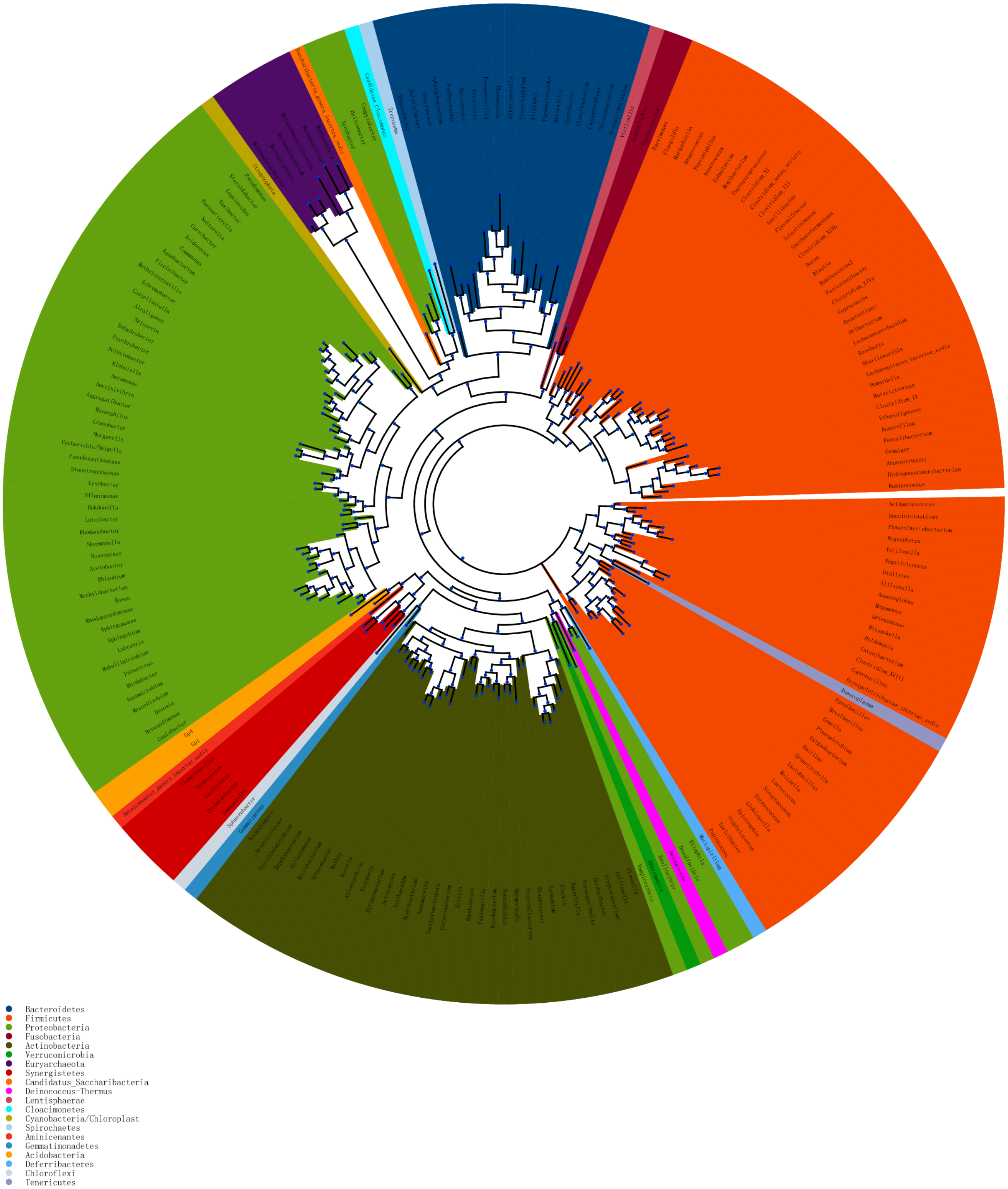


图6-9-1 物种进化树

注：分支的颜色表示对应的不同门水平分类。

# 七 Alpha多样性分析

## 7.1 单个样品多样性分析

Alpha多样性(Alpha diversity)是对单个样品中物种多样性的分析，包括observed species指数、chao1指数、shannon指数、simpson指数、PD_whole_tree指数以及ace指数。利用QIIME[16]-[17]软件计算样品的Alpha多样性指数的值，并做出相应的稀释曲线。

稀释曲线是利用已测得16S rDNA序列中已知的各种OTUs的相对比例，来计算抽取n个(n小于测得Reads序列总数)Reads时各Alpha多样性指数的期望值，然后根据一组n值(一般为一组小于总序列数的等差数列)与其相对应的Alpha多样性指数的期望值做出曲线来，并作出Alpha多样性指数的统计表格[18]。

observed_species指数表示实际观测到的otu数量；

goods_coverage指数表示测序深度:

$$C_{depth} = 1 - \frac{n_1}{N}$$

Cdepth：goods_coverage指数表示测序深度;

n1：只有含一条序列的OTU数目

N：为抽样中出现的总的序列数

参考网址：http://scikit-bio.org/docs/latest/generated/generated/skbio.diversity.alpha.goods_coverage.html

chao1指数用来估计样品所含OTU的总数，其公式为：

$$S_{chao1} = S_{obs} + \frac{n_1(n_1 - 1)}{2(n_2 + 1)}$$

Schao1：估计的OTU数量；

Sobs：实际观察到的OTU数量；

n1：只含一条序列的OTU的数量；

n2：含两条序列的OTU的数量；

参考网址：http://scikit-bio.org/docs/latest/generated/generated/skbio.diversity.alpha.chao1.html

shannon指数用来估算微生物群落的多样性，shannon值越大，多样性越高，其计算公式为：

$$H_{shannon} = -\sum_{i=1}^{S_{obs}} \frac{n_i}{N} ln \frac{n_i}{N}$$

Sobs：实际观察到的OTU数量；

ni：第i个OTU的序列数量；

N：所有的序列数

参考网址：http://scikit-bio.org/docs/latest/generated/generated/skbio.diversity.alpha.shannon.html

simpson指数和shannon指数一样用来估算微生物群落的多样性，simpson指数越大，多样性则越高，其计算公式为：

$$D_{simpson} = 1 - \sum_{i=1}^{S_{obs}} \frac{n_i(n_i - 1)}{N(N - 1)}$$

Sobs：实际观察到的OTU数量；

ni：第i个OTU的序列数量；

N：所有的序列数

参考网址：http://scikit-bio.org/docs/latest/generated/generated/skbio.diversity.alpha.simpson.html

ace指数是用来估计群落中含有OTU数目的指数，由Chao提出，是生态学中估计物种总数的常用指数之一，与Chao1的算法不同。

参考网址：http://scikit-bio.org/docs/latest/generated/generated/skbio.diversity.alpha.ace.html

软件平台：qiime/锐翌分析平台

结果目录： 07_Alpha_diversity\alpha_statistic.tsv\

表7-1-1 样品Alpha多样性统计结果

| sampleName | chao1 | goodsCoverage | observedSpecies | wholeTree | shannon | simpson | ace |
|---|---|---|---|---|---|---|---|
| A-24 | 173.36 | 1.00 | 139.00 | 11.17 | 3.84 | 0.86 | |
| A-25 | 236.33 | 1.00 | 171.00 | 12.54 | 4.21 | 0.89 | |
| A-26 | 371.38 | 1.00 | 290.00 | 21.58 | 5.43 | 0.96 | |
| A-9 | 266.56 | 1.00 | 190.00 | 13.68 | 4.28 | 0.90 | |
| A-20 | 195.00 | 1.00 | 166.00 | 11.70 | 4.12 | 0.88 | |
| A-21 | 168.00 | 1.00 | 141.00 | 11.44 | 4.09 | 0.89 | |
| A-22 | 400.60 | 1.00 | 304.00 | 22.09 | 5.62 | 0.96 | |
| A-23 | 236.11 | 1.00 | 210.00 | 14.09 | 4.74 | 0.92 | |
| B-14 | 242.50 | 1.00 | 200.00 | 13.32 | 5.32 | 0.96 | |
| B-15 | 266.25 | 1.00 | 225.00 | 14.98 | 4.06 | 0.81 | |

注：第一列Sample Name是样品名称；

第二列至最后一列是样品的不同的Alpha多样性指数的数值。

结果目录：alpha列表

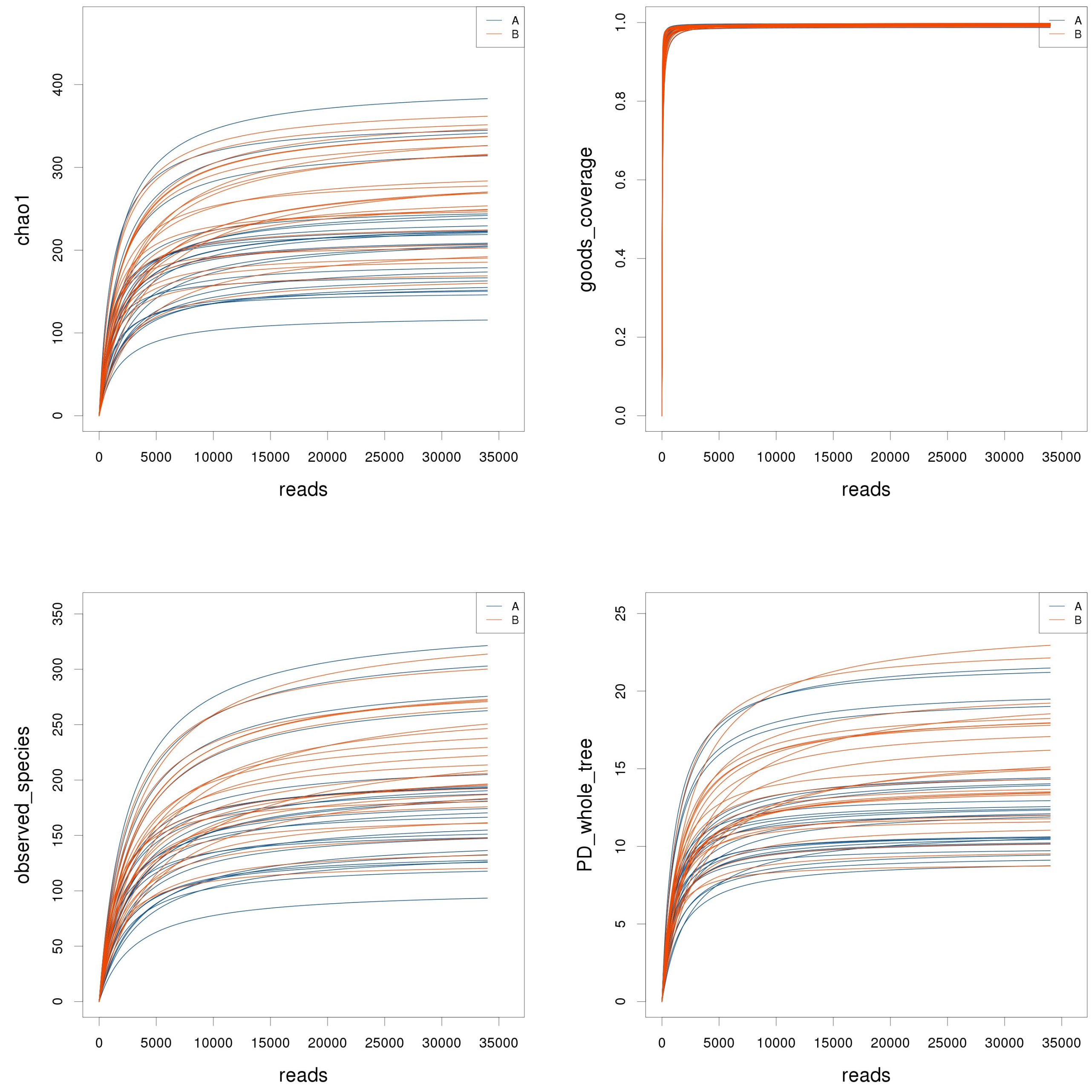

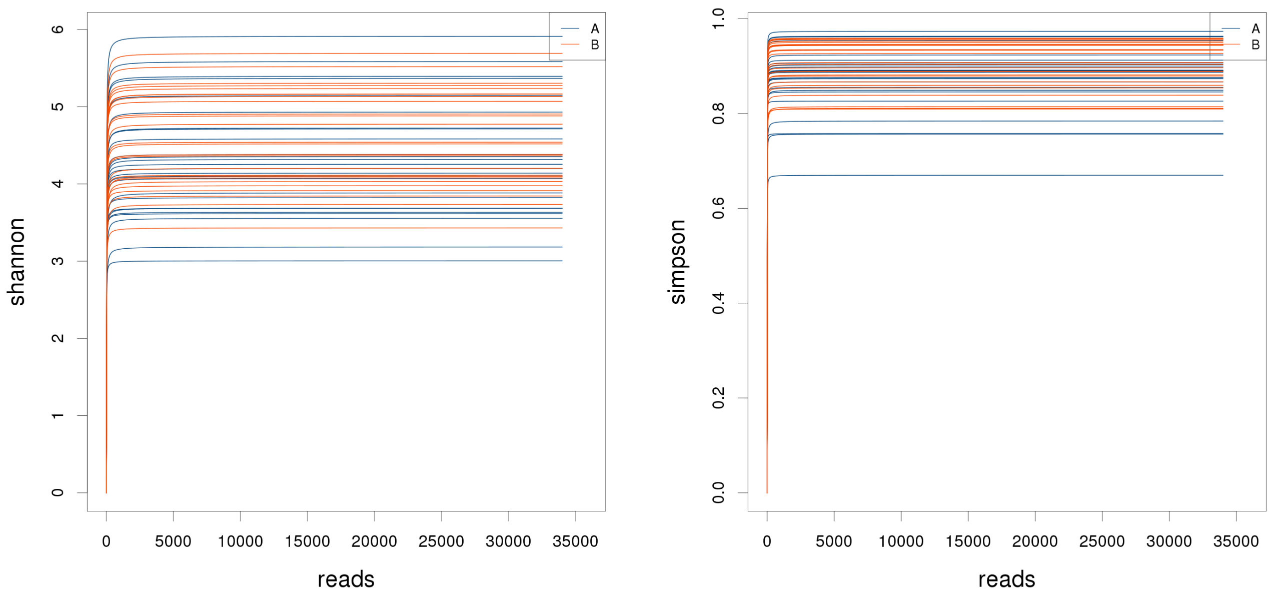


图7-1-1 样品物种丰度Alpha多样性指数稀释曲线图

注：横轴表示抽取的reads数量，纵轴表示相应Alpha多样性指数的值。图中一种颜色代表一组样品，测序条数不能覆盖样品时，曲线直线上升；测序条数增加到覆盖样品中的大部分微生物时，曲线呈现平滑趋势。

## 7.2 单个样品差异分析(每组内样品数≥3)

分别对Alpha diversity的各个指数进行秩和检验分析(若两组样品比较则使用R中的wilcox.test函数，若两组以上的样品比较则使用R中的kruskal.test函数（独立样本）或friedman.test（非独立样本）)，通过秩和检验筛选不同条件下的显著差异的Alpha Diversity指数。

软件平台：

结果目录： 07_Alpha_diversity \total_alpha_rare\ alpha_marker.tsv

结果目录：07_Alpha_diversity\group\box_plot\

表7-2-1 Alpha diversity指数差异检验

| sampleName | chao1 | goodsCoverage | observedSpecies | wholeTree | shannon | simpson | ace |
|---|---|---|---|---|---|---|---|
| p_value | 5.86e-03 | 6.37e-03 | 0.01 | 0.03 | 0.16 | 0.26 | |
| mean(A) | 243.27 | 1.00 | 198.27 | 14.50 | 4.34 | 0.87 | |
| mean(B) | 297.03 | 1.00 | 235.35 | 16.86 | 4.62 | 0.90 | |

注：第一行Alpha name是Alpha diversity指数；

第二行p-value是Alpha diversity指数对应的秩和检验的p值；

第三行至第4行分别为2组样品的均值；

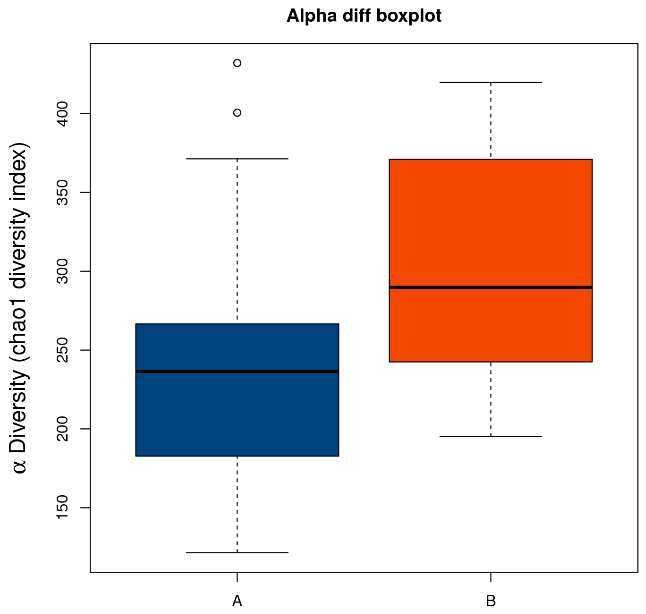
Alpha diff boxplot
α Diversity (chao1 diversity index)
150
200
250
300
350
400
A
B

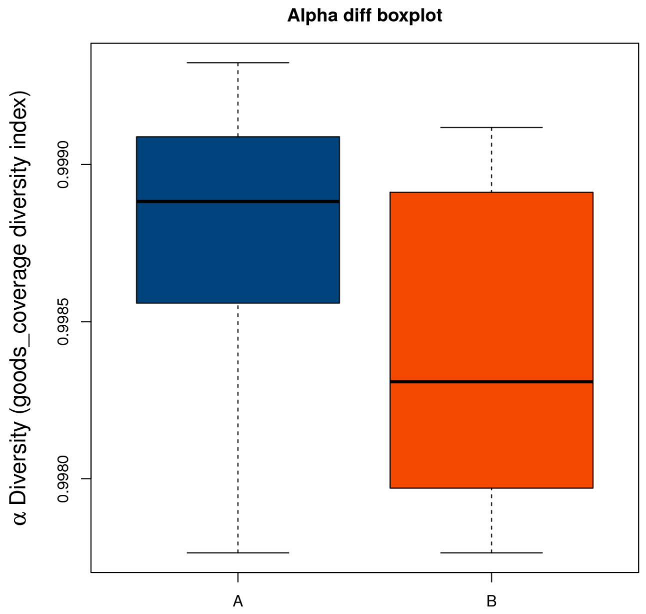
Alpha diff boxplot
α Diversity (goods_coverage diversity index)
0.9980
0.9985
0.9990
A
B

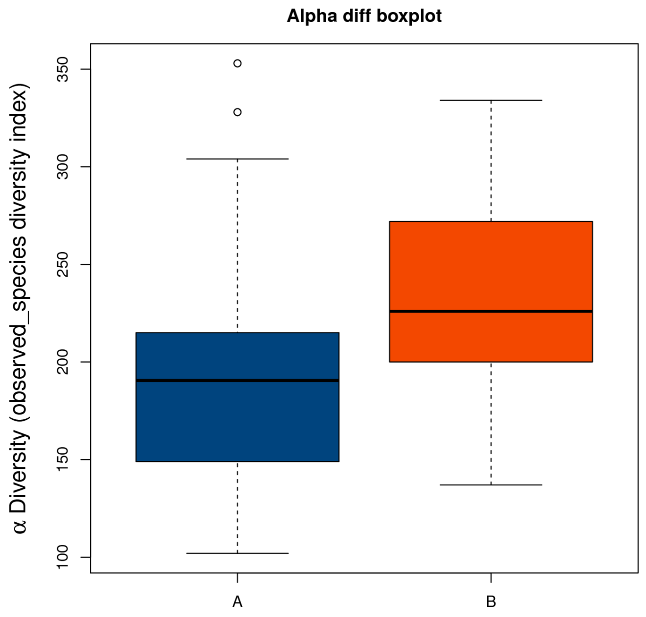
Alpha diff boxplot
α Diversity (observed_species diversity index)
100
150
200
250
300
350
A
B

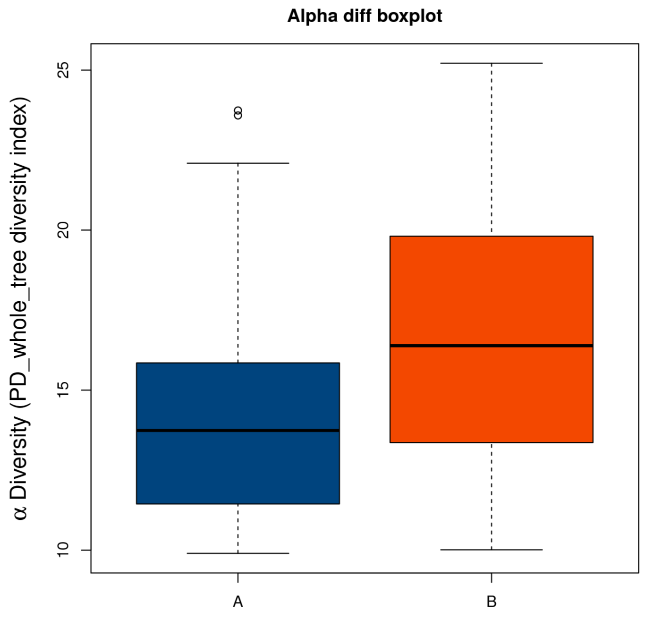
Alpha diff boxplot
α Diversity (PD_whole_tree diversity index)
10
15
20
25
A
B

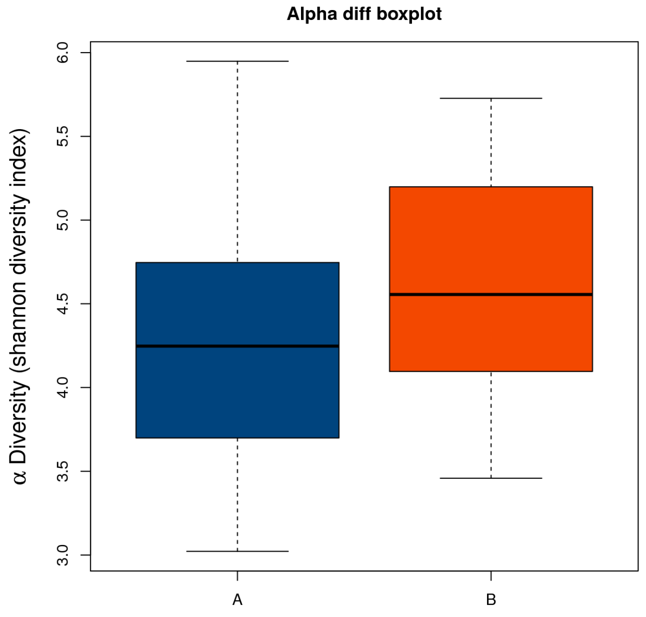
Alpha diff boxplot
α Diversity (shannon diversity index)
3.0
3.5
4.0
4.5
5.0
5.5
6.0
A
B

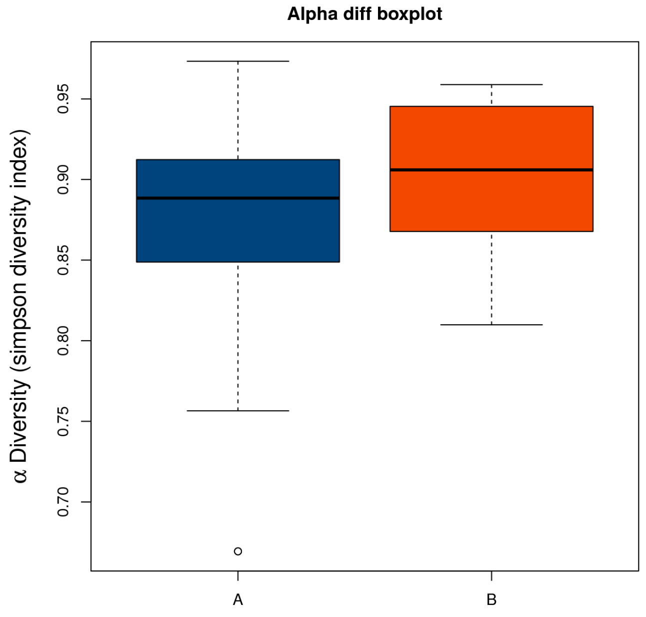
Alpha diff boxplot
α Diversity (simpson diversity index)
0.70
0.75
0.80
0.85
0.90
0.95
A
B

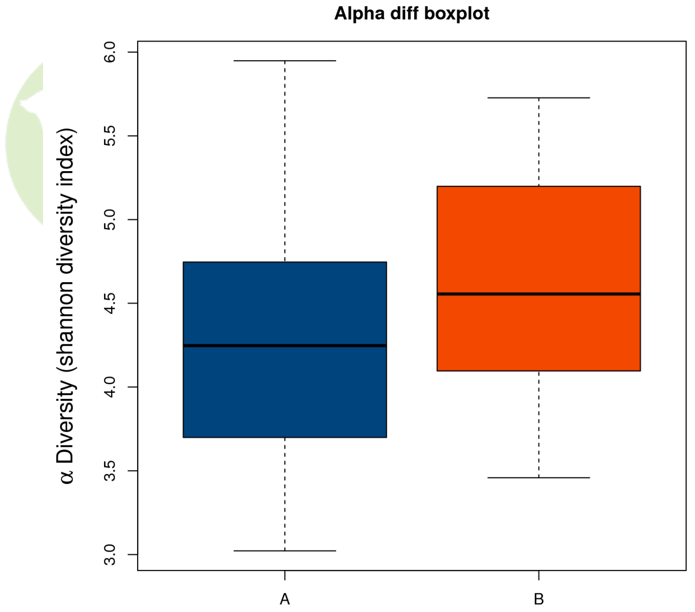


图7-2-1组间Alpha多样性盒形图：

注：横轴是分组名称，纵轴是不同分组下的alpha多样性指数的值。盒形图可以显示5个统计量(最小值，第一个四分位数，中位数，第三个中位数和最大值，及由下到上的5条线)，异常值以“o”标出。

# 八 Beta多样性分析

## 8.1 UniFrac热图分析（每组样品数≥4）

与Alpha多样性分析不同，Beta多样性（Beta Diversity）分析是用来比较一对样品在物种多样性方面存在的差异大小。

UniFrac是通过利用系统进化的信息来比较样品间的物种群落差异。其计算结果可以作为一种衡量Beta Diversity的指数，它考虑了物种间的进化距离，该指数越大表示样品间的差异越大。报告中给出的 UniFrac 结果分为加权 UniFrac (Weighted UniFrac)与非加权UniFrac(Unweighted UniFrac)两种，其中Weighted UniFrac考虑了序列的丰度，Unweighted UniFrac不考虑序列丰度。UniFrac 距离分布 Heatmap，通过对 UniFrac 结果的聚类，具有相似 Beta 多样性的样品聚类在一起，反应了样品间的相似性。

软件平台：qiime

结果目录：08_Beta_diversity\group\weighted_unifrac_otu_table.tsv

结果目录：08_Beta_diversity\group\unweighted_unifrac_otu_table.tsv

表8-1-1样品Beta Diversity统计表（Weighted Unifrac）

| sample Name | A-24 | A-25 | A-26 | A-9 | A-20 | A-21 | A-22 | A-23 |
|---|---|---|---|---|---|---|---|---|
| B-23 | 0.298809134157 | 0.412965645728 | 0.326558669886 | 0.303016268842 | 0.40583665224 | 0.238885210808 | 0.234472837322 | 0.23749347593 |
| B-4 | 0.41815486058 | 0.514316968788 | 0.333389691273 | 0.226161123651 | 0.393302395213 | 0.212595310159 | 0.173227370887 | 0.20946388304 |
| B-15 | 0.560387767781 | 0.707688773898 | 0.579193938095 | 0.345103161533 | 0.226378947802 | 0.410101638375 | 0.345261396955 | 0.49186067194 |
| B-26 | 0.464003571605 | 0.508442550676 | 0.39929461284 | 0.150637101791 | 0.343696752338 | 0.253316462184 | 0.226081325198 | 0.28690853954 |
| A-18 | 0.464160741696 | 0.565686115698 | 0.451993385023 | 0.369924055463 | 0.516930745536 | 0.383025460067 | 0.373373578163 | 0.35389303909 |
| B-20 | 0.506603800093 | 0.584510755685 | 0.474688284864 | 0.154999563595 | 0.354447494034 | 0.293909844952 | 0.277679569392 | 0.38565599729 |
| A-4 | 0.438638816688 | 0.534078255479 | 0.381811792083 | 0.189029611049 | 0.337240643772 | 0.309492202664 | 0.184586315104 | 0.30950031225 |
| B-2 | 0.445430761445 | 0.60905488411 | 0.505613740453 | 0.213478515511 | 0.294991370107 | 0.216490024621 | 0.285181121371 | 0.40444326583 |
| A-12 | 0.363555682241 | 0.477307117182 | 0.382988023883 | 0.258370713541 | 0.370609620266 | 0.168269502369 | 0.203919742451 | 0.25264744820 |
| A-14 | 0.451495090487 | 0.554834268719 | 0.343490435179 | 0.230617712745 | 0.42245666473 | 0.219152001285 | 0.229907180165 | 0.23844016104 |

表8-1-2样品Beta Diversity统计表（Unweighted Unifrac）

| sample Name | A-24 | A-25 | A-26 | A-9 | A-20 | A-21 | A-22 | A-23 |
|---|---|---|---|---|---|---|---|---|
| B-23 | 0.636830450713 | 0.61630437239 | 0.596715140095 | 0.57987055895 | 0.632265200398 | 0.606583219207 | 0.555291517876 | 0.62629494894 |
| B-4 | 0.606897273494 | 0.586691170144 | 0.527697758517 | 0.535660978651 | 0.594067123323 | 0.576078843499 | 0.474465242423 | 0.53212640454 |
| B-15 | 0.558308502657 | 0.550430296394 | 0.584868768464 | 0.518294341327 | 0.513403786317 | 0.544916989103 | 0.568890829555 | 0.50181052348 |
| B-26 | 0.488216453804 | 0.505955098616 | 0.609194862523 | 0.44361332111 | 0.499737117076 | 0.473744054997 | 0.562514360391 | 0.44836483329 |
| A-18 | 0.506293990731 | 0.521224596489 | 0.738709908847 | 0.602343828347 | 0.579720740951 | 0.560563328027 | 0.698243274448 | 0.63227819599 |
| B-20 | 0.567969336687 | 0.500143890113 | 0.564568630669 | 0.56353522593 | 0.562874976082 | 0.55574037194 | 0.526823003772 | 0.58446018795 |
| A-4 | 0.575737786564 | 0.588464616028 | 0.554937731449 | 0.419427461005 | 0.546755960408 | 0.511496472057 | 0.520518236826 | 0.47493299442 |
| B-2 | 0.408411438243 | 0.415913812392 | 0.653802040664 | 0.437988906587 | 0.440463002782 | 0.400816243414 | 0.62090638902 | 0.47664810165 |
| A-12 | 0.551269192686 | 0.505973040812 | 0.50710411835 | 0.50367527408 | 0.487554374682 | 0.519614264667 | 0.473317669452 | 0.46185574336 |
| A-14 | 0.533989164258 | 0.543909857564 | 0.578392630784 | 0.464072494162 | 0.474156539205 | 0.503113952604 | 0.556756937339 | 0.39932645705 |

注：表8-1-1为加权物种分类丰度信息计算得到的Beta Diversity统计结果;

表8-1-2为未加权物种分类丰度信息计算得到的Beta Diversity统计结果。

根据各样品差异性的统计结果，对样品进行聚类分析并计算样品间距离，以判断各样品物种组成的相似性。所有样品的聚类分析结果如下图所示：样品越靠近，说明两个样品的物种组成越相似。

软件平台：qiime

结果目录：08_Beta_diversity\group\heatmap\

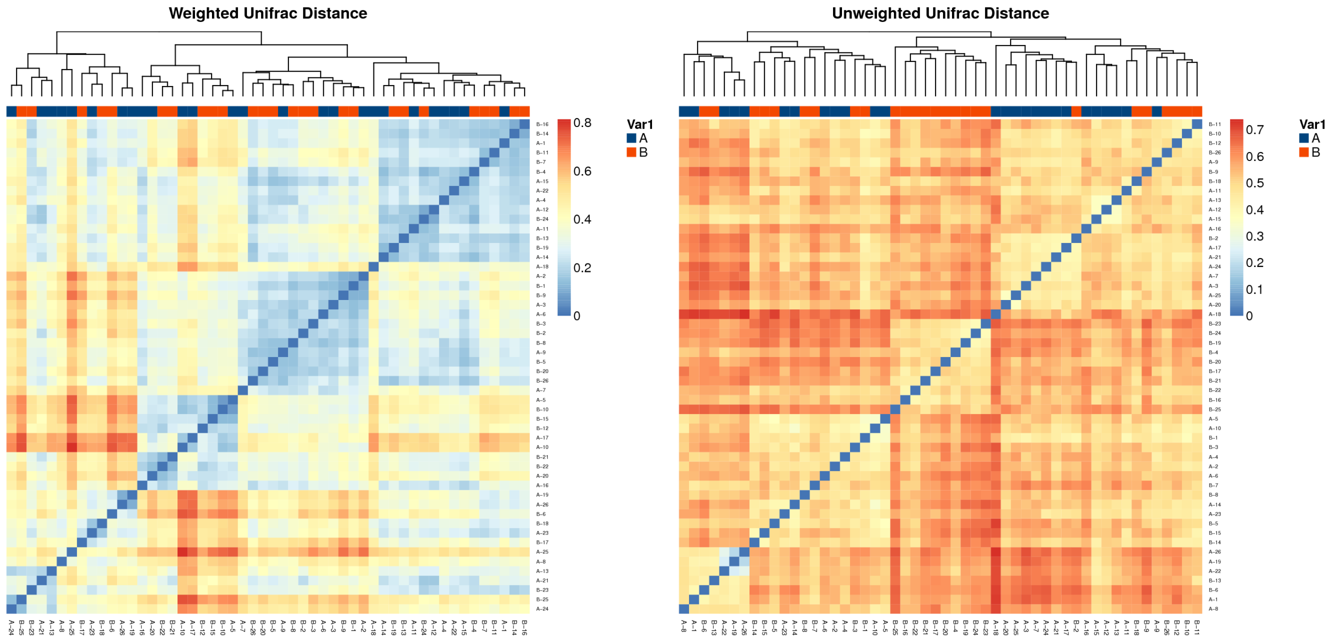


图8-1-3 Beta多样性heatmap（左图为Weighted，右图为Unweighted）

注：根据各样品差异性的统计结果，对样品进行聚类分析并计算样品间距离，以判断各样品物种组成的相似性。样品越靠近，说明两个样品的物种组成越相似。

## 8.2 Anosim分析（每组样品数≥5）

相似性分析Anosim分析是一种非参数检验，用来检验组间（两组或多组）的差异是否显著大于组内差异，从而判断分组是否有意义。首先利用 Unifrac 算法计算两两样品间的距离，然后将所有距离从小到大进行排序。

如果R值大于0，说明组间的差异大于组内差异，分组较为合理，反之R值若小于0，则说明组间差异小于组内差异，分组欠妥。P值越小组间差异越大。

软件平台：R vegan / 锐翌分析平台

结果目录：08_Beta_diversity\group\anosim\

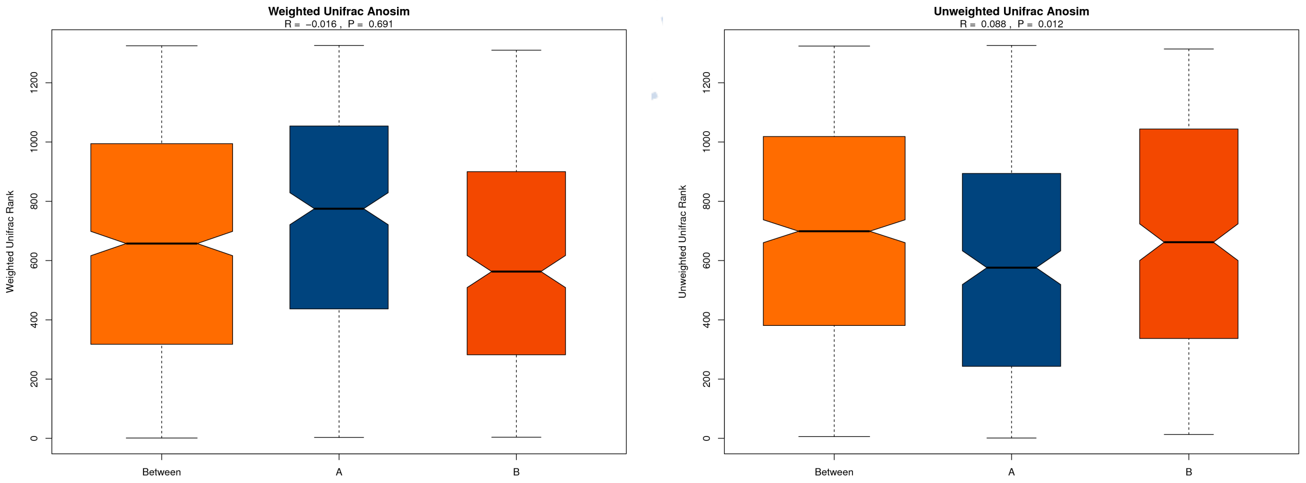


图8-2-1 Anosim分析图（左图为Weighted_Unifrac结果，右图为Unweighted_Unifrac结果）

注：横坐标表示所有样品 (Between) 以及每个分组 (Control、LDC)，纵坐标表示unifrac距离的秩。Between组相对与其他每个分组的秩较高时，则表明组间差异大于组内差异。R介于(-1, 1)之间，R大于0，说明组间差异显著；R小于0，说明组内差异大于组间差异，统计分析的可信度用P表示，P小于0.05 表示统计具有显著性。

## 8.3 物种MRPP分析（每组样品数≥5）

MRPP组间差异分析是用于分析组间微生物群落结构的差异是否显著的一种分析方法，通常与PCA、PCoA、NMDS等降维图配合使用。

软件平台：R/vegan

结果目录：08_Beta_diversity\group\mrpp\mrpp.tsv

表8-3-1 MRPP组间差异分析

| group | a | observeDelta | expectDelta | significance |
| --- | --- | --- | --- | --- |
| weighted_unifrac | -0.00493601494115969 | 0.366467500792972 | 0.364667496581292 | 0.763 |
| unweighted_unifrac | 0.010237750706364 | 0.526467495491278 | 0.531913089094884 | 0.008 |

注：Group：分为weighted_unifrac、unweighted_unifrac；

A：A统计量；

ObserveDelta：Unifrac距离指数对应的ObserveDelta值；

ExpectDelta：Unifrac距离指数对应的ExpectDelta值；

Significance：Unifrac距离指数对应的显著性p值；

A值大于0说明组间差异大于组内差异，A值小于0说明组内差异大于组间差异；ObserveDelta值越小说明组内差异越小；ExpectDelta值越小说明组间差异越小；Significance小于0.05说明差异显著；

## 8.4 PCoA分析（每组样品数≥5）

为了进一步展示样品间物种多样性差异，使用主坐标分析(Principal Coordinates Analysis，PCoA)的方法展示各个样品间的差异大小[19]-[21]。如下图给出了PCoA对样品间物种多样性的分析结果，如果两个样品距离较近，则表示这两个样品的物种组成较相近。

软件平台：锐翌分析平台

结果目录：08_Beta_diversity\group\pcoa\

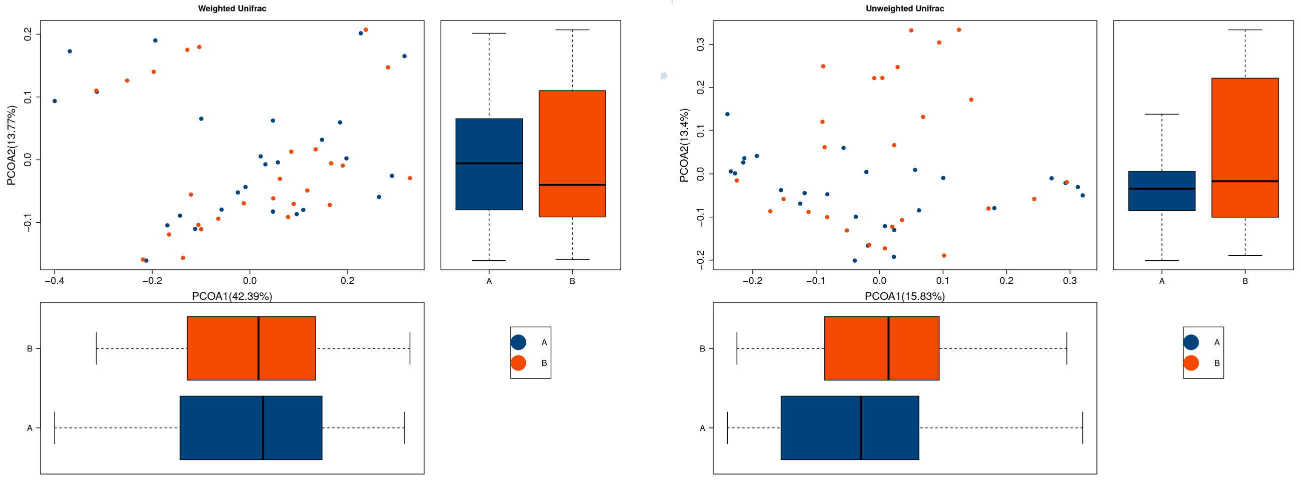


图8-4-1 PCoA分析图（左图为Weighted_UniFrac结果，右图为Unweighted_UniFrac结果）

注：横坐标表示第一主坐标，括号中的百分比则表示第一主坐标对样品差异的贡献率；纵坐标表示第二主坐标，括号中的百分比表示第二主坐标对样品差异的贡献率。PCoA是一种研究数据相似性或者差异性的可视化方法，它没有改变样品点之间的相互位置关系，只改变了坐标系统。图中各点分别表示各个样品，不同颜色代表样品属于不同的分组。

## 8.5 非度量多维尺度分析(NMDS)(总样品数≥5)

为了进一步展示样品间物种多样性差异，使用NMDS分析(Nonmetric Multidimensional Scaling)的方法展示各个样品间的差异大小[22]。如下图给出了NMDS对样品间物种多样性的分析结果，如果两个样品距离较近，则表示这两个样品的物种组成较相近。

软件平台：R vegan

结果目录：08_Beta_diversity\group\nmds\

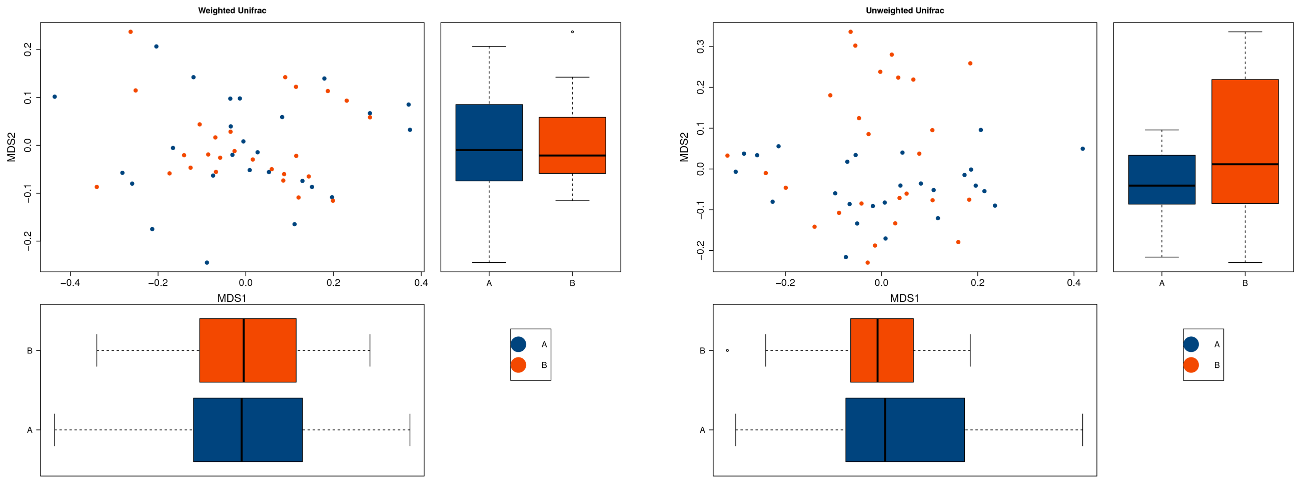


图8-5-1 NMDS分析图（左图为Weighted_UniFrac结果，右图为Unweighted_UniFrac结果）

注：横轴和纵轴表示基于进化或者数量距离矩阵的数值在二维表中成图。不同颜色代表样品属于不同的分组，各点分别表示各个样品，点与点之间的距离表示差异程度。

## 8.6 UPGMA层次聚类（总样品数≥5）

UPGMA(Unweighted pair group method with arithmetic mean，非加权组平均法)常用来解决分类问题的一种聚类分析方法，其假设的前提是：在进化过程中所有核苷酸(氨基酸)的变异率相同。通过UPGMA构建系统进化树[23]，使得结果可视化，可以直观显示不同的环境样本中微生物进化的差异程度。

软件平台：锐翌分析平台

结果目录：08_Beta_diversity\group\cluster\

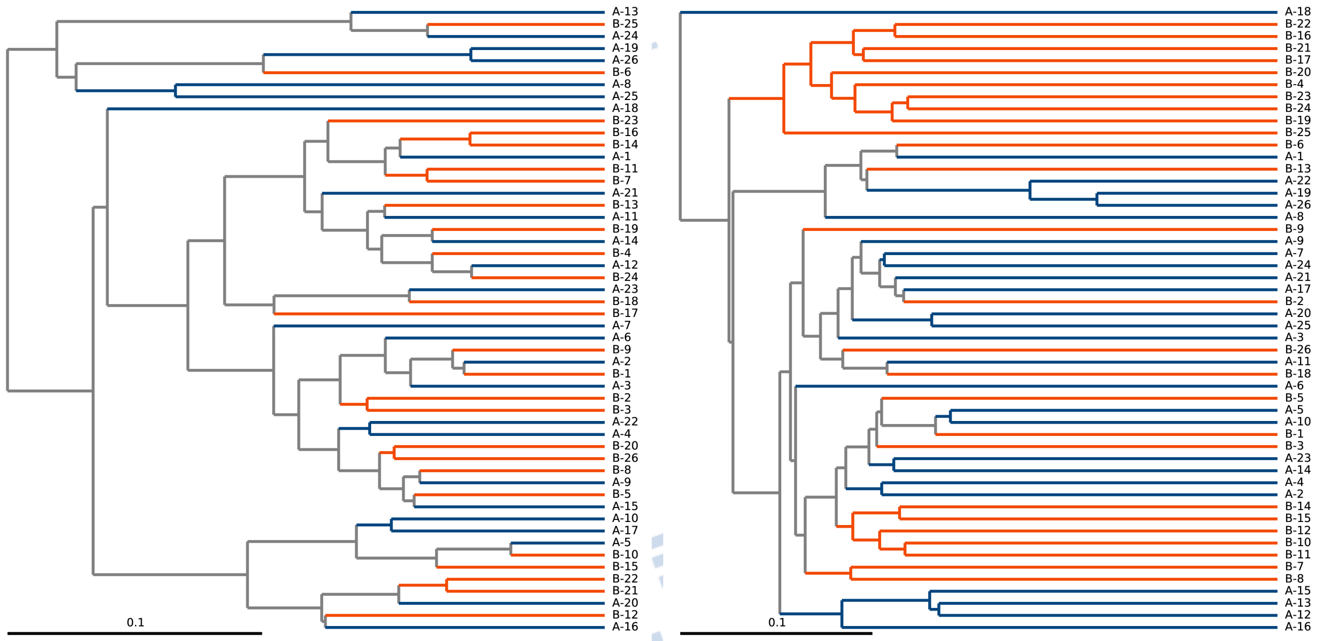


图8-6-1 UPGMA层次聚类分析（左图为Weighted_UniFrac结果，右图为Unweighted_UniFrac结果）

注：树枝不同颜色代表不同的分组。

# 九 显著性差异分析

## 9.1 LDA EffectSize（LEfSe）分析(每组样品数≥3)

LDA EffectSize(LEfSe分析)：LEfSe采用线性判别分析 (LDA) 来估算每个组分(物种)丰度对差异效果影响的大小，找出对样品划分产生显著性差异影响的群落或物种。LEfSe分析强调统计意义和生物相关性[24]。

软件平台：LEfSe

结果目录：09_diff_analysis\group\LEfSe\

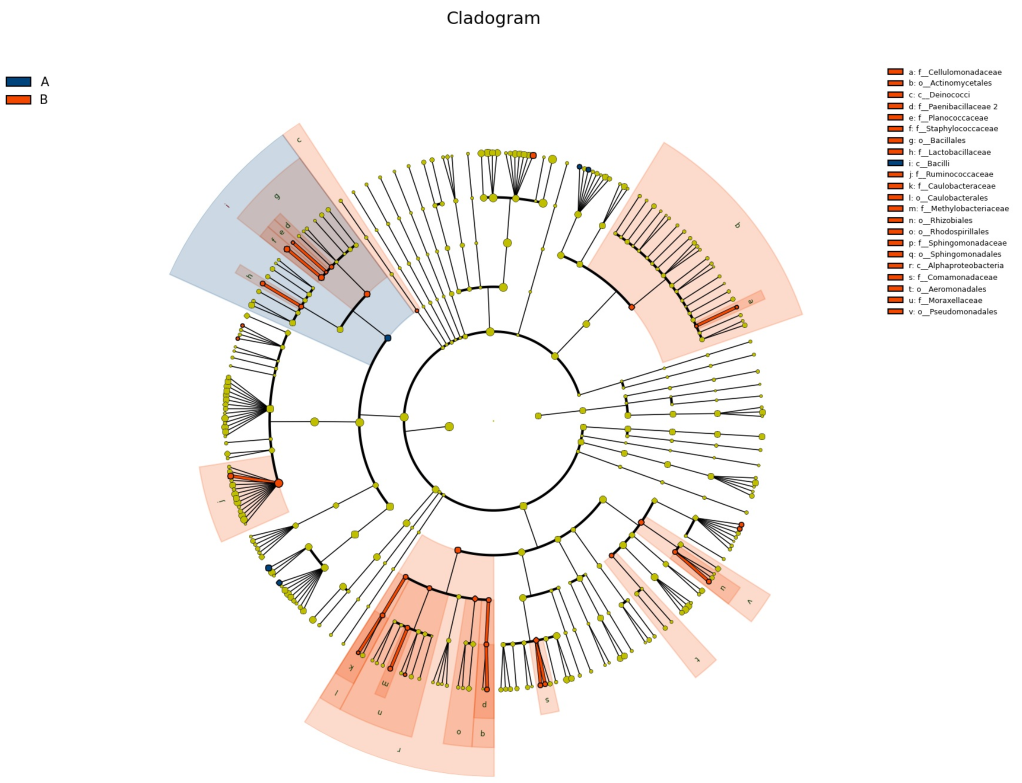

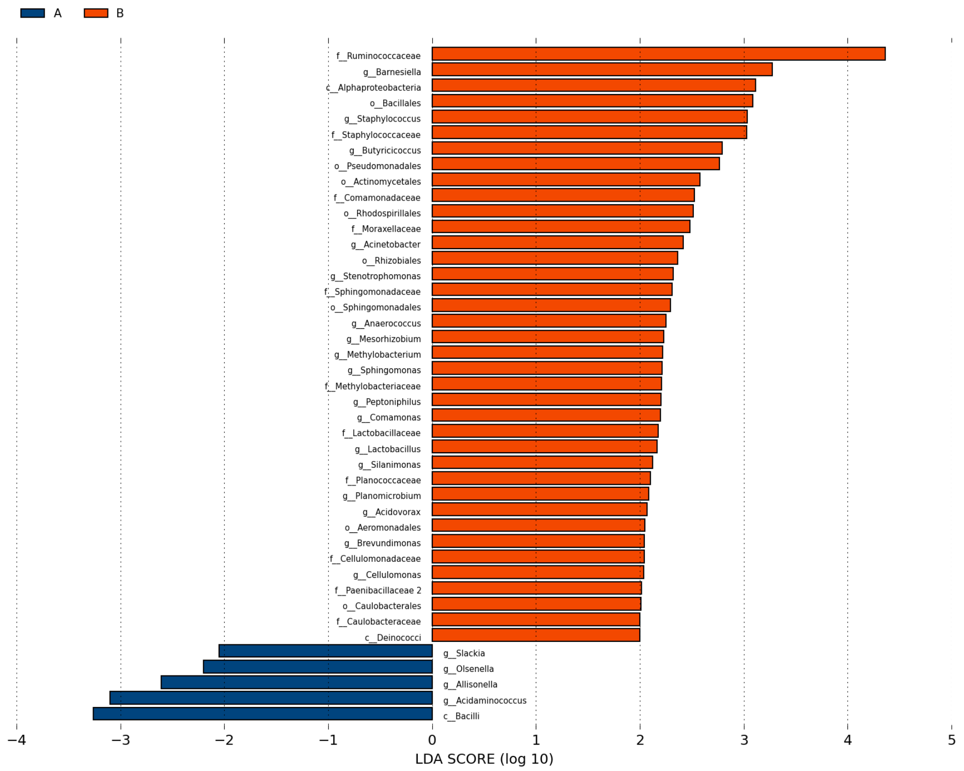


图9-1-1 LEfSe分析图

注：上图为聚类树，不同颜色表示不同分组，不同颜色的节点表示在该颜色所代表的分组中起到重要作用的微生物群。黄色节点表示的是在不同分组中没有起到重要作用的微生物类群。图中英文字母所表示的物种名称在右侧图例中进行展示。下图是统计不同分组中有显著作用的微生物类群通过LDA(线性回归分析)后得到的LDA分值。（LDA阀值2）。

## 9.2 组间差异分析（PCA:每组样品数≥3;Heatmap:每组样品数≥3;Boxplot:每组样品数≥5）

OTU水平的差异分析

使用秩和检验的方法对不同分组之间进行显著性差异分析，以找出对组间划分产生显著性差异影响的物种。本分析对于两组间的差异分析采用R语言stats包的wilcox.test函数，对于两组以上的组间差异分析采用R语言stats包的kruskal.test函数（独立样本）或friedman.test函数（非独立样本）。

软件平台：锐翌分析平台

结果目录：09_diff_analysis\group\genus_diff\

结果目录：09_diff_analysis\group\otu_diff\

结果目录：09_diff_analysis\group\taxall_diff\

下表是在不同组样品间有显著差异的OTUs（p ＜ 0.05），共114个，如表9-2-1显示。结果文件 otu_diff.marker.filt.tsv

表9-2-1差异显著OTU列表

锐翌基因
REALGENE

| taxonname | mean(A) | fdr | mean(B) | pvalue |
|---|---|---|---|---|
| denovo34 | 0.00838817489940672 | 0.277754348651046 | 0.00880383845248607 | 0.0229312155749057 |
| denovo93 | 0.00245811187468207 | 0.351408622566861 | 0.00678987468903982 | 0.0319132701608707 |
| denovo73 | 0.00579186016824975 | 0.366803172829691 | 0.0001561375783062 | 0.0403428702764816 |
| denovo80 | 0.00160252014491245 | 0.264419948029007 | 0.0031755007479812 | 0.0198313047925085 |
| denovo50 | 0.000367134305746935 | 0.366803172829691 | 0.00439295186531922 | 0.0396366311980543 |
| denovo678 | 0.00141895299203897 | 0.305099097102695 | 0.00251719095836878 | 0.0264482189438869 |
| denovo106 | 0.000788072776990735 | 0.25357285767089 | 0.00293707444597558 | 0.0154394206425 |
| denovo105 | 0.00263112919118805 | 0.366803172829691 | 0 | 0.0413813854920809 |
| denovo101 | 0.00197281940157282 | 0.235662233647209 | 0.000406168700323742 | 0.0138624843321888 |
| denovo103 | 4.21993454881538e-06 | 0.0842597058670557 | 0.00198969913976778 | 0.000997060788152484 |

注：第一列是OTU名称；

第二列至第4列分别为2组样品的均值；

最后一列是秩和检验的p值；

为了直观地对这些OTUs进行展示，绘制对应图形如下：

软件平台：锐翌分析平台

结果目录：09_diff_analysis\group\otu_diff\heatmap\

结果目录：09_diff_analysis\group\otu_diff\boxplot\

结果目录：09_diff_analysis\group\otu_diff\pca\

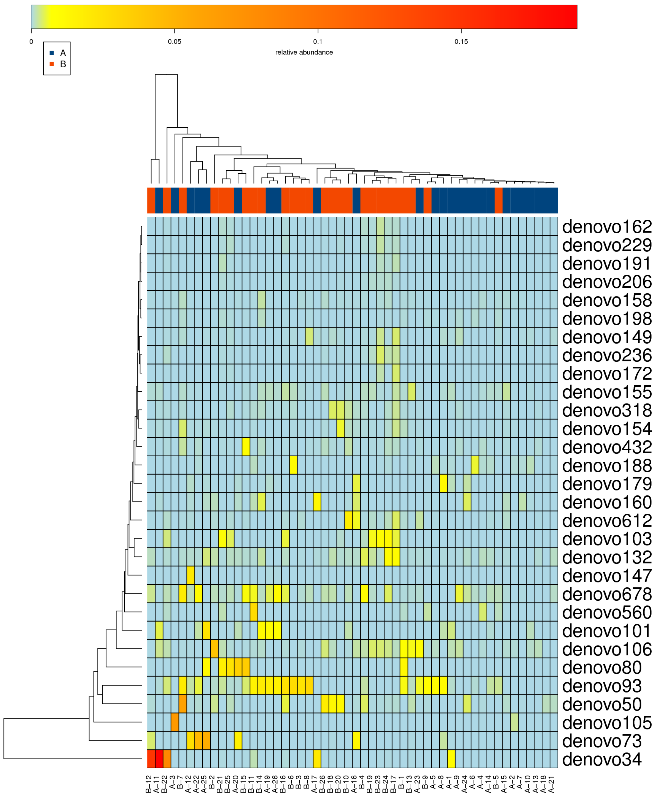


图9-2-1 差异OTU的Heatmap结果展示

注：横向聚类表示该物种在各样品丰度相似情况，距离越近，枝长越短，说明两物种在各样品间的组成越相似。纵向聚类表示所有物种在不同样品间表达的相似情况，距离越近，枝长越短，说明样品的物种组成及丰度越相似。

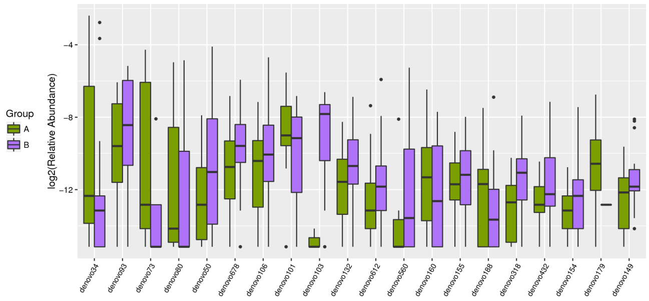


图9-2-2 差异OTU的Boxplot结果展示

注：横坐标是OTU的名称，纵坐标是相对丰度log2的值，不同颜色代表不同分组。

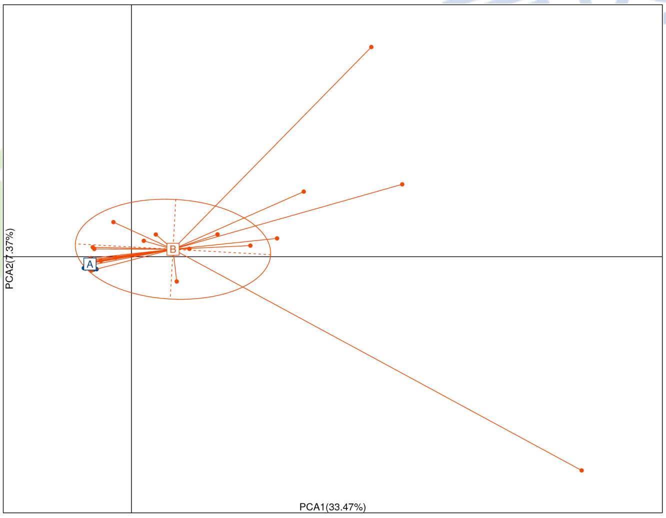


图9-2-3 差异OTU的PCA结果展示

注：横坐标表示第一主成分，百分比则表示第一主成分对样品差异的贡献值；纵坐标表示第二主成分，百分比表示第二主成分对样品差异的贡献值；图中的每个点表示一个样品，同一个组的样品使用同一种颜色表示。

属水平的差异分析

通过秩和检验可以找出在不同组间有明显差异(p ＜ 0.05)的属如下（共54个属）。 结果表格 genus_diff.marker.filt.tsv

表9-2-2差异显著物种列表

| taxonname | mean(A) | fdr | mean(B) | pvalue |
|---|---|---|---|---|
| g__Acetobacter | 0 | 0.0701328256292681 | 1.37147872836308e-05 | 0.0105539689053753 |
| g__Achromobacter | 2.10996727440769e-06 | 0.0263099195411526 | 5.16941982229462e-05 | 0.00251222353143543 |
| g__Acidaminococcus | 0.00248870640016082 | 0.0179427222838408 | 5.06392145859461e-05 | 0.00080556699146196 |
| g__Acidovorax | 0 | 0.0167659067724563 | 0.000200446891068558 | 0.000569715278675699 |
| g__Acinetobacter | 3.16495091161154e-06 | 0.0179427222838408 | 0.000492677358574485 | 0.00107345305445804 |
| g__Aeromonas | 0 | 0.0701328256292681 | 7.27938709672385e-05 | 0.0105216964385158 |
| g__Allisonella | 0.000967419995315977 | 0.128180709291695 | 0.000305945254789377 | 0.0248894581148923 |
| g__Anaerococcus | 1.05498363720385e-06 | 0.0701328256292681 | 2.00446891068846e-05 | 0.0101467811486314 |
| g__Aquabacterium | 2.10996727440769e-06 | 0.0799494410693677 | 5.90790836833769e-05 | 0.0124193306515523 |
| g__Bacillus | 0 | 0.0167659067724563 | 0.000114993216455185 | 0.0002597307947677 |

注：第一列是物种名称；

第二列至第4列分别为2组样品的均值；

最后一列是秩和检验的p值。

为了直观地对这些差异属进行展示，绘制对应图形如下：

软件平台：锐翌分析平台

结果目录：09_diff_analysis\group\genus_diff\heatmap\

结果目录：09_diff_analysis\group\genus_diff\boxplot\

结果目录：09_diff_analysis\group\genus_diff\pca\

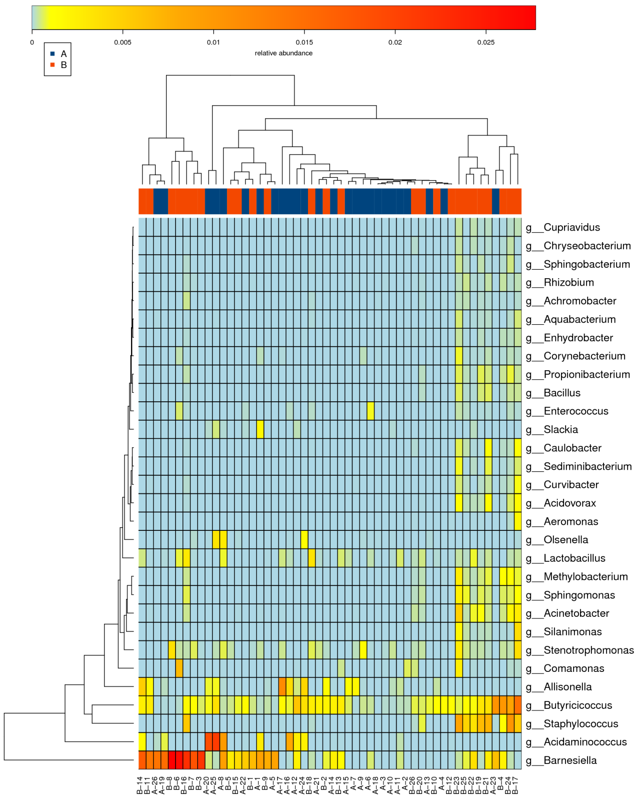


图9-2-4 属水平差异物种的Heatmap结果展示

注：横向聚类表示该物种在各样品丰度相似情况，距离越近，枝长越短，说明两物种在各样品间的组成越相似。纵向聚类表示所有物种在不同样品间表达的相似情况，距离越近，枝长越短，说明样品的物种组成及丰度越相似。

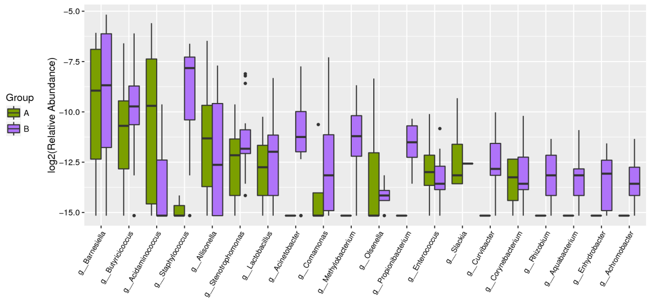


图9-2-5 属水平差异物种的Boxplot结果展示

注：横坐标是名称，纵坐标是相对丰度log2的值，不同颜色代表不同分组。

锐翌基因 REALGENE

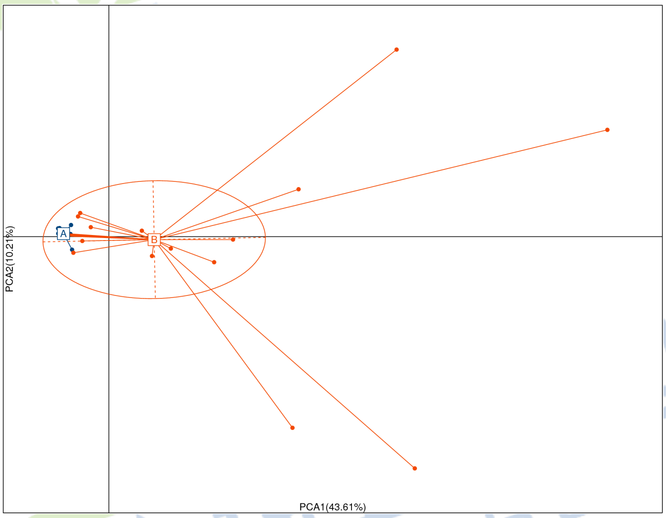


图9-2-6 属水平差异物种的PCA结果展示

注：横坐标表示第一主成分，百分比则表示第一主成分对样品差异的贡献值；纵坐标表示第二主成分，百分比表示第二主成分对样品差异的贡献值；图中的每个点表示一个样品，同一个组的样品使用同一种颜色表示。

所有水平的差异分析

通过秩和检验可以找出在不同组间有明显差异(p ＜ 0.05)的物种(包含所有水平)如下 （共115个）。结果表格 taxall_diff.marker.filt.tsv

表9-2-3所有水平差异显著物种列表

| taxonname | mean(A) | fdr | mean(B) | pvalue |
|---|---|---|---|---|
| c__Alphaproteobacteria | 0.00014242279102255 | 0.0223248480149689 | 0.00270708801306241 | 0.00174744809864817 |
| c__Bacilli | 0.00515043011683778 | 0.104758046256412 | 0.00451321999995717 | 0.0191164317986154 |
| c__Deferribacteres | 0 | 0.160450466389106 | 5.27491818601923e-06 | 0.0413001238645938 |
| c__Deinococci | 0 | 0.160450466389106 | 1.26598036464577e-05 | 0.0413001238645938 |
| c__Flavobacteriia | 3.16495091161154e-06 | 0.0238600550119456 | 7.80687891530423e-05 | 0.00249043829302583 |
| c__Sphingobacteriia | 0 | 0.0167058938545663 | 0.000164577447403588 | 0.000569060165245067 |
| f__Acetobacteraceae | 0 | 0.0638870668502453 | 4.32543291253346e-05 | 0.0105539689053753 |
| f__Aeromonadaceae | 0 | 0.0638870668502453 | 7.27938709672385e-05 | 0.0105216964385158 |
| f__Bacillaceae 1 | 0 | 0.0167058938545663 | 0.000114993216455185 | 0.0002597307947677 |
| f__Brevibacteriaceae | 0 | 0.0167058938545663 | 2.53196072928731e-05 | 0.00118314097983963 |

注:第一列是物种名称;

第二列至第4列分别为2组样品的均值;

最后一列是秩和检验的p值。

为了直观地对这些差异物种进行展示，绘制对应图形如下：

软件平台：锐翌分析平台

结果目录：09_diff_analysis\group\taxall_diff\heatmap\

结果目录：09_diff_analysis\group\taxall_diff\boxplot\

结果目录：09_diff_analysis\group\taxall_diff\pca\

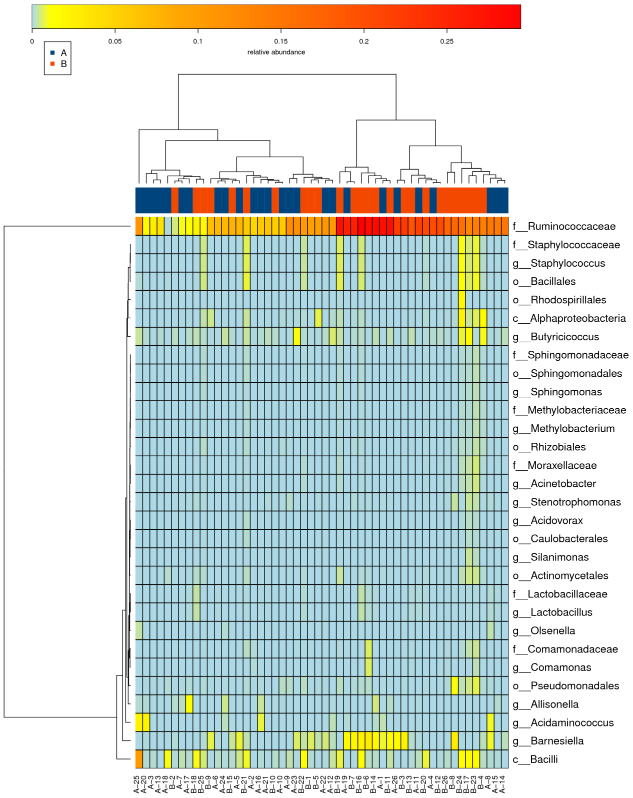


图9-2-7 所有水平差异物种的Heatmap结果展示

注：横向聚类表示该物种在各样品丰度相似情况，距离越近，枝长越短，说明两物种在各样品间的组成越相似。纵向聚类表示所有物种在不同样品间表达的相似情况，距离越近，枝长越短，说明样品的物种组成及丰度越相似。

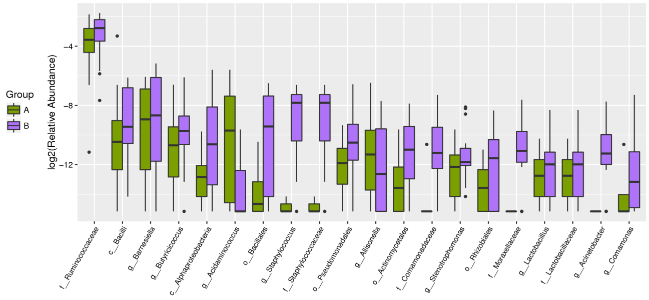


图9-2-8 所有水平差异物种的Boxplot结果展示

注：横坐标是名称，纵坐标是相对丰度log2的值，不同颜色代表不同分组。

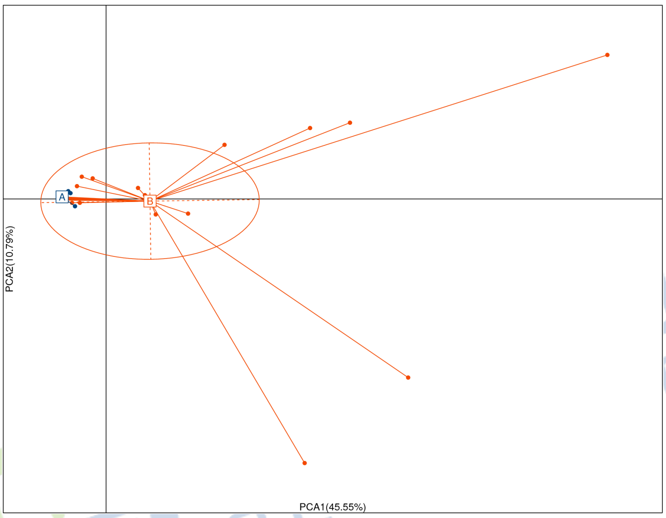


图9-2-9 所有水平差异物种的PCA结果展示

注：横坐标表示第一主成分，百分比则表示第一主成分对样品差异的贡献值；纵坐标表示第二主成分，百分比表示

第二主成分对样品差异的贡献值；图中的每个点表示一个样品，同一个组的样品使用同一种颜色表示。

## 9.3 优势物种Spearman相关系数分析（每组样品数≥3）

使用LEfSe在各水平上或秩和检验在属水平上（或某一特定水平），选择丰度前30的差异物种，通过R软件的corrplot包绘制优势物种之间spearman相关性热图，并通过该热图可以发现优势物种之间重要的模式与关系。

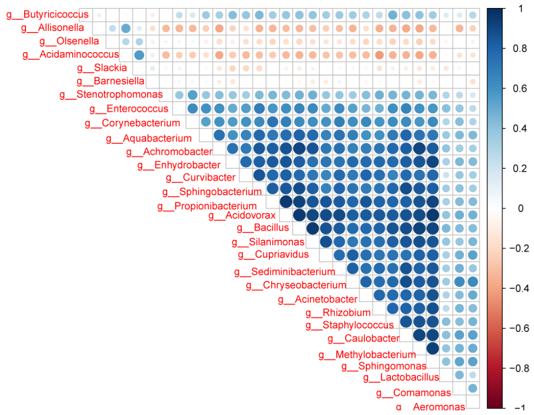


图9-3-1 丰度前30的差异物种相关性系数分析

注：在属水平上，通过秩和检验得到差异物种，计算丰度前30的差异物种之间的相关系。右边蓝色表示正相关，红色表示负相关。颜色越深，表明物种之间的相关性越强。

# 十、个性化分析

# 备注：

# 结果目录：

RESULTS

├──04_data_statistics [Reads]

| | length_distrubution.pdf [reads长度]

| | length_distrubution.png [reads长度]

| | length_distrubution.tsv [reads长度统计数据]

| | pick_otu_summary.tsv [reads长度]

| |

| └──alpha

| chao1.pdf [Alpha多样性chao1指数]

| chao1.png [chao1指数]

| chao1.txt [chao1指数的数据]

| observed_species.pdf [observed_species指数]

| observed_species.png [observed_species指数]

| observed_species.txt [observed_species指数的数据]

|

├──05_OTU_analysis [OTU分析]

| ├──all [所有]

| | | otu_downsize_stat.tsv [OTU抽平结果]

| | | otu_statistic.tsv [OTU统计]

| | | otu_table.tsv [OTU丰度表]

| | | profile_tree.tsv [层次分类丰度表]

| | | rep_set.fna [OTU代表序列]

```
| | | tax_assignment.tsv [OTU注释文件]
| | |
| | ├─specaccum [物种累积曲线]
| | | specaccum.pdf
| | | specaccum.png
| | |
| | └─wf_taxa_summary [OTU注释到各个水平]
| | otu_table_L2.txt [门水平]
| | otu_table_L3.txt [纲水平]
| | otu_table_L4.txt [目水平]
| | otu_table_L5.txt [科水平]
| | otu_table_L6.txt [属水平]
| |
| └─group [group分组一]
| | otu_downsize_stat.tsv [OTU抽平结果]
| | otu_statistic.tsv [OTU数量统计]
| | otu_table.tsv [OTU丰度表]
| | profile_tree.tsv [层次分类丰度表]
| | tax_assignment.tsv [OTU注释文件]
| |
| ├─core_otu [共有OTU]
| | core_otu.pdf [共有OTU]
| | core_otu.png [共有OTU]
| | core_otu.txt [共有OTU数据]
| | for_plot.txt [画图数据]
| |
| ├─flower [花瓣图]
| | for_plot.txt [画图数据]
| | flower.png
| |
| ├─otu_pca [PCA图]
| | otu_pca.pdf
| | otu_pca.png
| |
| ├─venn [维恩图]
| | for_plot.txt
| | venn.png
| |
| └─wf_taxa_summary [OTU注释到各个水平]
| otu_table_all.txt [所有水平]
| otu_table_L1.txt [界水平]
| otu_table_L2.txt [门水平]
```

```
| otu_table_L3.txt [纲水平]
| otu_table_L4.txt [目水平]
| otu_table_L5.txt [科水平]
| otu_table_L6.txt [属水平]
|
├─06_classification_abundance_analysis
| ├─all [所有]
| | | krona.html [注释结果可视化]
| | |
| | ├─bar_plot [物种注释柱状图]
| | | ├─class [纲水平]
| | | | bar_plot.pdf [柱状图]
| | | | bar_plot.png
| | | | for_plot.txt [画图数据]
| | | |
| | | ├─family [科水平]
| | | | bar_plot.pdf
| | | | bar_plot.png
| | | | for_plot.txt
| | | |
| | | ├─genus [属水平]
| | | | bar_plot.pdf
| | | | bar_plot.png
| | | | for_plot.txt
| | | |
| | | ├─order [目水平]
| | | | bar_plot.pdf
| | | | bar_plot.png
| | | | for_plot.txt
| | | |
| | | └─phylum [门水平]
| | | bar_plot.pdf
| | | bar_plot.png
| | | for_plot.txt
| | |
| | ├─rank_abundance [rank_abundance曲线图]
| | | rank_abundance.pdf
| | | rank_abundance.png
| | |
| | └─tax_star [星形图]
| | for_star_plot.txt
| | tax_star.pdf
```

```
| | tax_star.png
| |
| └─group [分组1]
| | krona.html [注释结果可视化]
| |
| ├─bar_plot [物种注释柱状图]
| | ├─class [纲水平]
| | | bar_plot.pdf [柱状图]
| | | bar_plot.png [柱状图]
| | | for_plot.txt [画图数据]
| | |
| | ├─family [科水平]
| | | bar_plot.pdf [柱状图]
| | | bar_plot.png
| | | for_plot.txt
| | |
| | ├─genus [属水平]
| | | bar_plot.pdf [柱状图]
| | | bar_plot.png
| | | for_plot.txt
| | |
| | ├─order [目水平]
| | | bar_plot.pdf [柱状图]
| | | bar_plot.png
| | | for_plot.txt
| | |
| | └─phylum [门水平]
| | bar_plot.pdf [柱状图]
| | bar_plot.png
| | for_plot.txt
| |
| ├─heatmap [热图]
| | for_plot.txt
| | heatmap.pdf
| | heatmap.png
| |
| ├─phylo_tree [进化树]
| | tax_phylo.nwk
| |
| ├─tax_bar_tree [物种聚类]
| | bar_tree.pdf
| | bar_tree.png
```

```
|  |  for_plot.txt
|  |
|  └─tax_tree [物种分类树]
| tax_ass_modified.txt
| tax_tree.nwk
|
├─07_Alpha_diversity [alpha多样性]
|  ├─group [分组一]
|  |  | alpha_marker.tsv [alpha显著差异marker]
|  |  | alpha_statistic.tsv [alpha多样性统计]
|  |  | chao1.pdf [chao1指数]
|  |  | chao1.png []
|  |  | chao1.txt []
|  |  | goods_coverage.pdf [goods_coverage指数]
|  |  | goods_coverage.png []
|  |  | goods_coverage.txt []
|  |  | observed_species.pdf [observed_species指数]
|  |  | observed_species.png []
|  |  | observed_species.txt []
|  |  | PD_whole_tree.pdf [PD_whole_tree指数]
|  |  | PD_whole_tree.png []
|  |  | PD_whole_tree.txt []
|  |  | shannon.pdf [shannon指数]
|  |  | shannon.png []
|  |  | shannon.txt []
|  |  | simpson.pdf [simpson指数]
|  |  | simpson.png []
|  |  | simpson.txt []
|  |  |
|  |  └─box_plot [盒型图]
|  | chao1.boxplot.pdf [chao1指数]
|  | chao1.boxplot.png []
|  | goods_coverage.boxplot.pdf [goods_coverage指数]
|  | goods_coverage.boxplot.png []
|  | observed_species.boxplot.pdf [observed_species指数]
|  | observed_species.boxplot.png []
|  | PD_whole_tree.boxplot.pdf [PD_whole_tree指数]
|  | PD_whole_tree.boxplot.png []
|  | shannon.boxplot.pdf [shannon指数]
|  | shannon.boxplot.png []
|  | simpson.boxplot.pdf [simpson指数]
|  | simpson.boxplot.png []
```

```
│  │
│  └─total_alpha_rare [总的alpha多样性]
│  alpha_statistic.tsv [alpha多样性统计]
│  chao1.pdf [chao1指数]
│  chao1.png []
│  chao1.txt []
│  goods_coverage.pdf [goods_coverage指数]
│  goods_coverage.png []
│  goods_coverage.txt []
│  observed_species.pdf
│  observed_species.png [observed_species指数]
│  observed_species.txt
│  PD_whole_tree.pdf
│  PD_whole_tree.png [PD_whole_tree指数]
│  PD_whole_tree.txt
│  shannon.pdf
│  shannon.png [shannon指数]
│  shannon.txt
│  simpson.pdf
│  simpson.png [simpson指数]
│  simpson.txt
│
├─08_Beta_diversity [beta多样性]
│  └─group [分组一]
│  │  unweighted_unifrac_otu_table.tsv [未加权unifranc距离表]
│  │  weighted_unifrac_otu_table.tsv [加权unifrac距离表]
│  │
│  ├─anosim [anosim结果]
│  │  unweighted_unifrac.anosim.pdf
│  │  unweighted_unifrac.anosim.png
│  │  weighted_unifrac.anosim.pdf
│  │  weighted_unifrac.anosim.png
│  │
│  ├─cluster [聚类结果]
│  │  mapfile.txt
│  │  unweighted_unifrac_cluster.pdf
│  │  unweighted_unifrac_cluster.png
│  │  unweighted_unifrac_otu_table.txt
│  │  weighted_unifrac_cluster.pdf
│  │  weighted_unifrac_cluster.png
│  │  weighted_unifrac_otu_table.txt
│  │
```

```
| ├─heatmap [热图]
| | unweighted_unifrac.heatmap.pdf
| | unweighted_unifrac.heatmap.png
| | weighted_unifrac.heatmap.pdf
| | weighted_unifrac.heatmap.png
| |
| ├─nmds [nmds结果]
| | unweighted_unifrac.nmds.pdf
| | unweighted_unifrac.nmds.png
| | weighted_unifrac.nmds.pdf
| | weighted_unifrac.nmds.png
| |
| └─pcoa [pcoa结果]
| unweighted_unifrac.pcoa.pdf
| unweighted_unifrac.pcoa.png
| weighted_unifrac.pcoa.pdf
| weighted_unifrac.pcoa.png
|
└─09_diff_analysis [差异分析]
└─group [分组一]
├─genus_diff [属水平差异]
| | genus_diff.marker.filt.tsv [过滤后的差异maker]
| | genus_diff.marker.tsv [差异maker]
| |
| ├─boxplot [盒型图]
| | diff_boxplot.for_plot_top_20.txt []
| | diff_boxplot.pdf []
| | diff_boxplot.png []
| |
| ├─heatmap [热图]
| | heatmap.pdf
| | heatmap.png
| |
| └─pca [pca图]
| diff_pca.pdf
| diff_pca.png
|
├─LEfSe [lEfSe结果]
| LDA.cladogram.pdf
| LDA.cladogram.png
| LDA.pdf
| LDA.png
```

```
│ otu_table_for_lefse.txt
│
├─otu_diff [OTU水平差异]
│ │ otu_diff.marker.filt.tsv
│ │ otu_diff.marker.tsv
│ │
│ ├─boxplot [盒型图]
│ │ diff_boxplot.for_plot_top_20.txt
│ │ diff_boxplot.pdf
│ │ diff_boxplot.png
│ │
│ ├─heatmap [热图]
│ │ heatmap.pdf
│ │ heatmap.png
│ │
│ └─pca [pca图]
│ diff_pca.pdf
│ diff_pca.png
│
└─taxall_diff [所有水平的差异]
│ taxall_diff.marker.filt.tsv
│ taxall_diff.marker.tsv
│
├─boxplot [盒型图]
│ diff_boxplot.for_plot_top_20.txt
│ diff_boxplot.pdf
│ diff_boxplot.png
│
├─heatmap [热图]
│ heatmap.pdf
│ heatmap.png
│
└─pca [pca图]
diff_pca.pdf
diff_pca.png
```

# 文件打开或浏览方法

生物信息分析的相关文件均为Linux系统下的文件。Windows用户，一般文本文件可以使用写字板打开。Unix/Linux用户可以使用more或less命令查看该文本文件内容；推荐使用开源文本编辑器gedit for win32版本(http://projects.gnome.org/gedit/)。

图片打开方式：数据中可能包含部分图像文件，一般图像文件后缀名为.png、.pdf、tiff等，对于图像文件，Windows用户可以使用图片浏览器打开，Linux/Unix用户使用display命令打开。

表格打开方式：Linx下的表格均为制表符(Tab)分割的文本，为了便于阅读，建议使用excel或openoffice等办公软件用表格形式打开，打开时请用“Tab分割”方式。

当文件比较大时，打开文件可能导致Windows系统死机，建议使用性能较好的计算机或者使用更适合处理大量数据的Unix/Linux系统打开。

# 参考文献

[1] PANDAseq: paired-end assembler for illumina sequences.Andre P Masella, Andrea K Bartram, Jakub M Truszkowski, Daniel G Brown and Josh D Neufeld. [J] BMC Bioinformatics, 2012.

[2] UCHIME improves sensitivity and speed of chimera detection Edgar, R.C, Haas,BJ. et al. [J] Bioinformatics, 2011.

[3] UPARSE: Highly accurate OTU sequences from microbial amplicon reads. Edgar, R.C. [J] Nature Methods, 2013.

[4] Metagenomic sequencing reveals altered metabolic pathways in the oral microbiota of sailors during a long sea voyage Zheng W, Zhang Z, Liu C, et al. [J]. Scientific reports, 2015.

[5] Baleen whales host a unique gut microbiome with similarities to both carnivores and herbivores Sanders J G, Beichman A C, Roman J, et al. [J] Nature communications, 2015.

[6] Ribosomal Database Project: data and tools for high throughput rRNA analysis. Cole, J. R., Q. Wang, J. A. Fish, B. Chai, D. M. McGarrell, Y. Sun, C. T. Brown, A. Porras-Alfaro, [J] Nucleic Acids Research, 2014.

[7] Naïve Bayesian Classifier for Rapid Assignment of rRNA Sequences into the New Bacterial Taxonomy.Wang, Q,G. M. Garrity,J. M. Tiedje, and J. R. Cole. [J] Appl Environ Microbiol, 2007.

[8] Comparative analysis of the gastrointestinal microbial communities of bar-headed goose (Anser indicus) in different breeding patterns by high-throughput sequencing .Wang W, Cao J, Li J R, et al. [J]. Microbiological research, 2016.

[9] Changes in the composition and diversity of microbial communities during anaerobic nitrate reduction and Fe (II) oxidation at circumneutral pH in paddy soil Li X, Zhang W, Liu T, et al. [J] Soil Biology and Biochemistry, 2016.

[10] Biogeography of the intestinal mucosal and lumenal microbiome in the rhesus macaque Yasuda K, Oh K, Ren B, et al. [J] Cell host & microbe, 2015.

[11] Effect of bacterial communities on the formation of cast iron corrosion tubercles in reclaimed water Jin J, Wu G, Guan Y. [J] Water research, 2015.

[12] Comparative analysis of the gastrointestinal microbial communities of bar-headed goose (Anser indicus) in different breeding patterns by high-throughput sequencing Wang W, Cao J, Li J R, et al. [J]. Microbiological research, 2016.

[13] Metagenomic sequencing reveals altered metabolic pathways in the oral microbiota of sailors during a long sea voyage. Zheng W, Zhang Z, Liu C, et al. [J]. Scientific reports, 2015.

[14] Illumina MiSeq sequencing reveals diverse microbial communities of activated sludge systems stimulated by different aromatics for indigo biosynthesis from indole .Zhang X, Qu Y, Ma Q, et al. [J]. PloS one, 2015.

[15] Phylogenetic and functional gene structure shifts of the oral microbiomes in periodontitis patients Li Y, He J, He Z, et al. [J] The ISME journal, 2014.

[16] QIIME allows analysis of high-throughput community sequencing data. Caporaso JG, Kuczynski J. et al. [J] Nature Methods, 2010.

[17] Bacterial diversity in aquatic and other environments:what 16S rDNA libraries can tell us. Paul FK, Josephine Y A. [J] FEMS Microbiol.Ecol, 2004.

[18] Bacterioplankton community shifts associated with epipelagic and mesopelagic waters in the Southern Ocean Yu Z, Yang J, Liu L, et al. [J] Scientific reports, 2015.

[19] Structural modulation of gut microbiota during alleviation of type 2 diabetes with a Chinese herbal formula.Xu J, Lian F, Zhao L, et al. [J]The ISME Journal, 2015.

[20] Biogeography of the intestinal mucosal and lumenal microbiome in the rhesus macaque .Yasuda K, Oh K, Ren B, et al. [J]Cell host & microbe, 2015.

[21] Deciphering chicken gut microbial dynamics based on high-throughput 16S rRNA metagenomics analyses Shaufi M A M, Sieo C C, Chong C W, et al. [J] Gut pathogens, 2015.

[22] Vineyard soil bacterial diversity and composition revealed by 16S rRNA genes: differentiation by geographic features Burns K N, Kluepfel D A, Strauss S L, et al. [J] Soil Biology and Biochemistry, 2015.

[23] Phylogenetic and functional gene structure shifts of the oral microbiomes in periodontitis patients Li Y, He J, He Z, et al. [J]. The ISME journal, 2014.

[24] Metagenomic biomarker discovery and explanation. Segata, N., Izard, J., Waldron, L., Gevers, D., Miropolsky, L., Garrett, W.S., and Huttenhower, C. [J] Genome Biol, 2011.

[25] Structural modulation of gut microbiota during alleviation of type 2 diabetes with a Chinese herbal formula. Xu J, Lian F, Zhao L, et al. [J] The ISME Journal, 2015.

[26] STAMP: Statistical analysis of taxonomic and functional profiles. Parks DH, Tyson GW, Hugenholtz P, et al. [J] Bioinformatics, 2014.

[27] Predictive functional profiling of microbial communities using 16S rRNA marker gene sequences. Langille, M. G.I.*; Zaneveld, J.*; Caporaso, J. G.; McDonald, D.; Knights, D.; a Reyes, J.; Clemente, J. C.; Burkepile, D. E.; Vega Thurber, R. L.; Knight, R.; Beiko, R. G.; and Huttenhower, C. [J] Nature Biotechnology, 2013.
