# Supplementary figures and images for: Gut microbiota induces high platelet response in patients with ST segment elevation myocardial infarction after ticagrelor treatment
Source: eLife. 2022 Mar 8;11:e70240. doi: 10.7554/eLife.70240 (PMC8903831; doi:10.7554/eLife.70240)

001


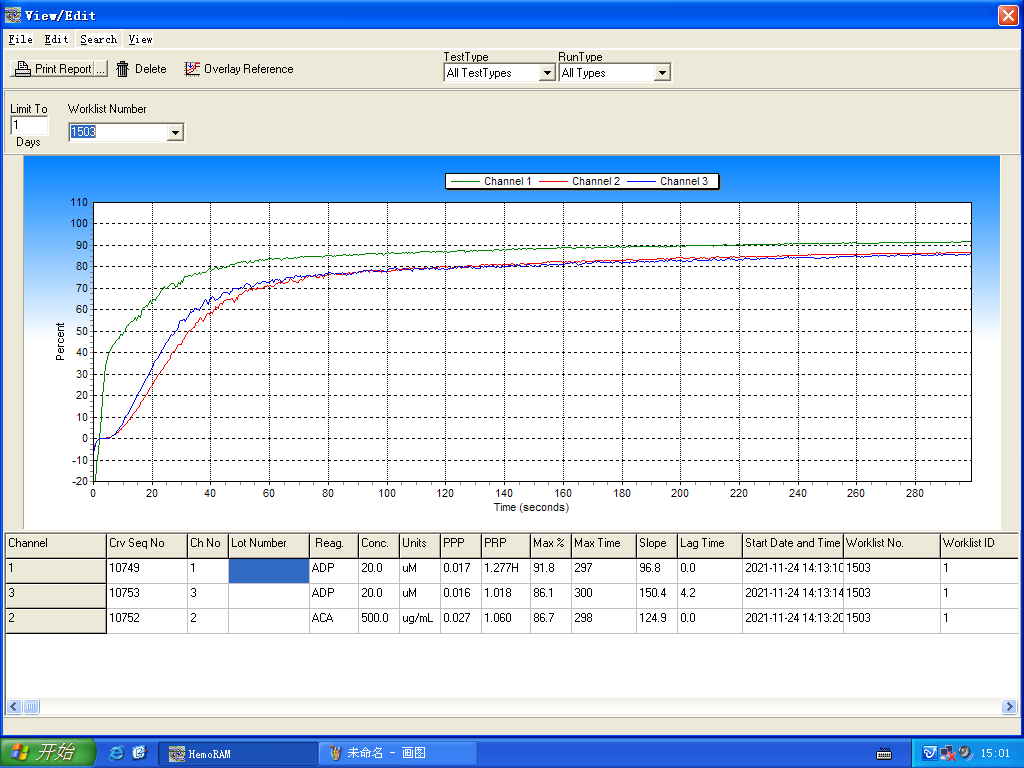


002


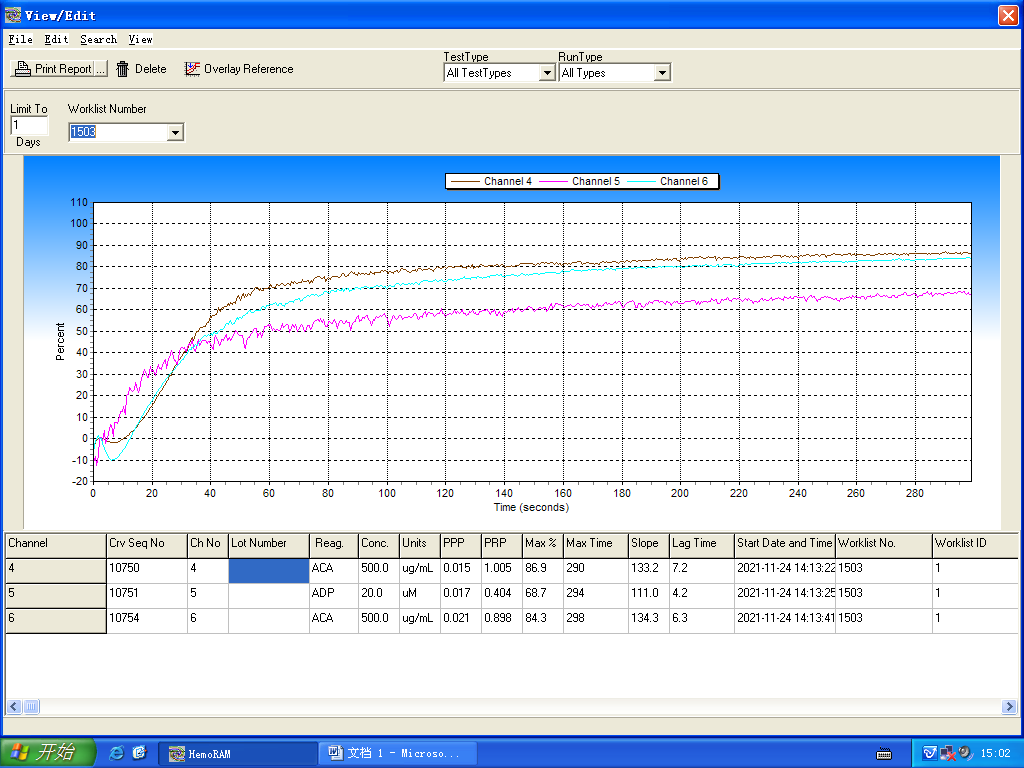


003


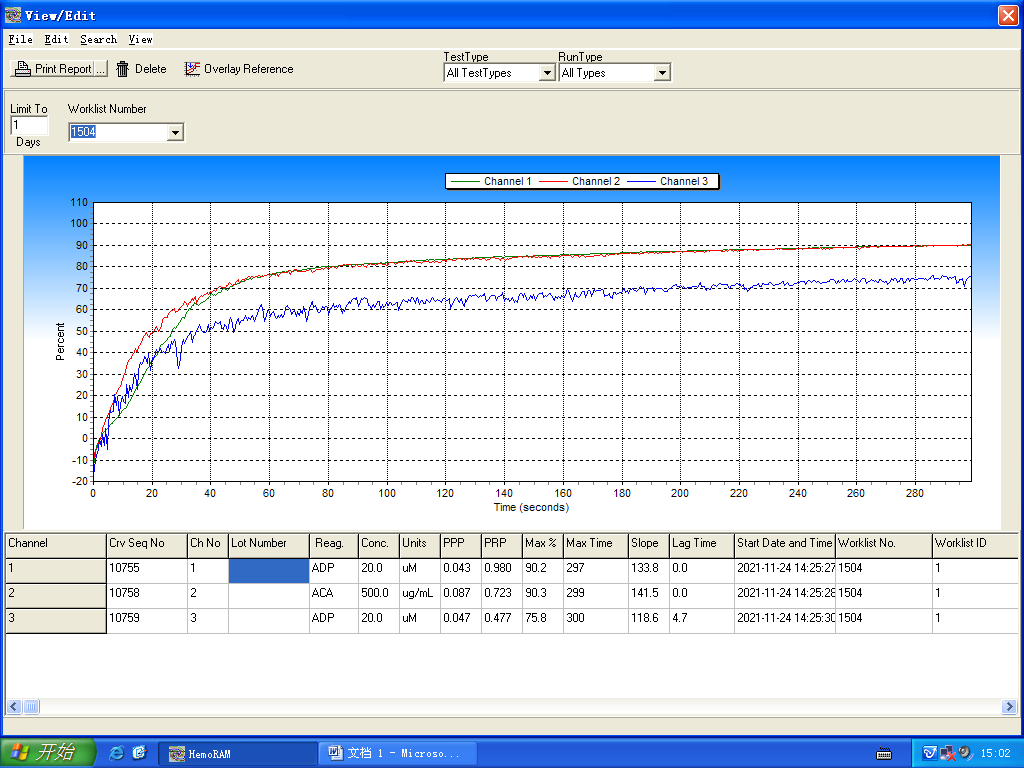


101


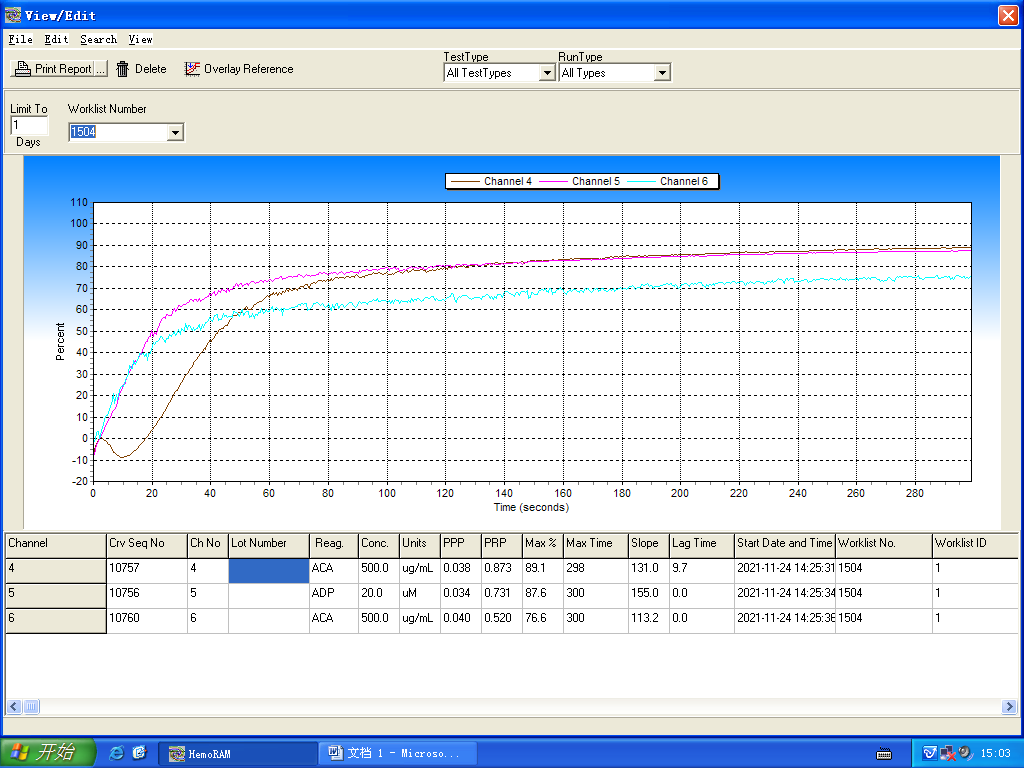


102


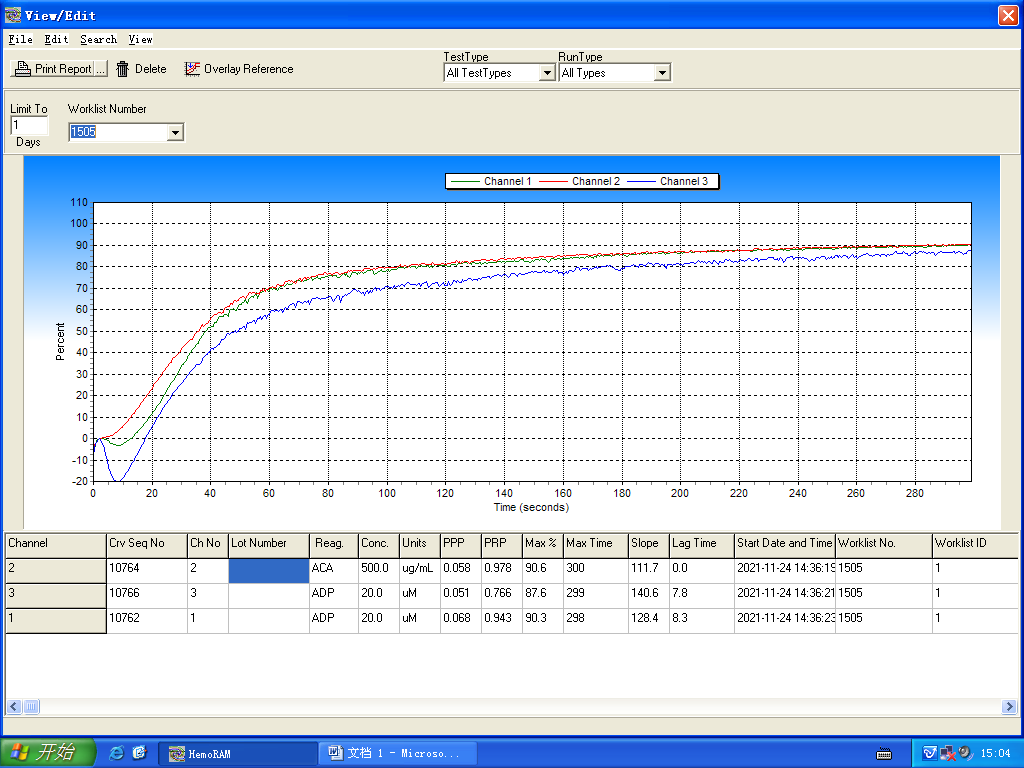


103


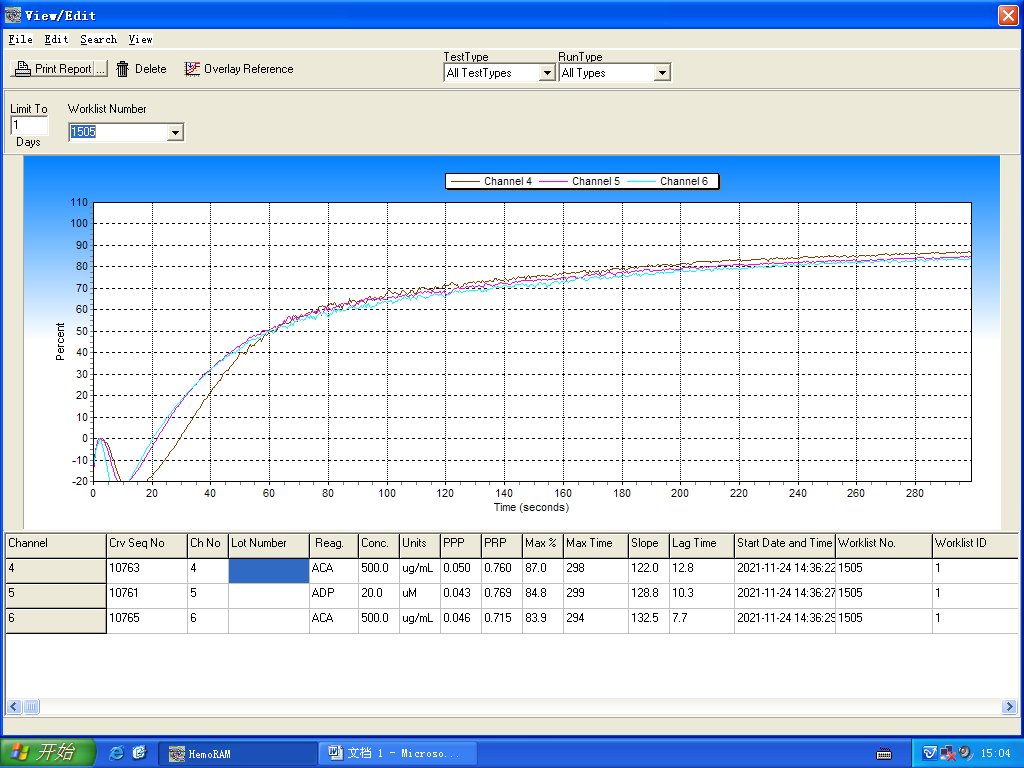


401


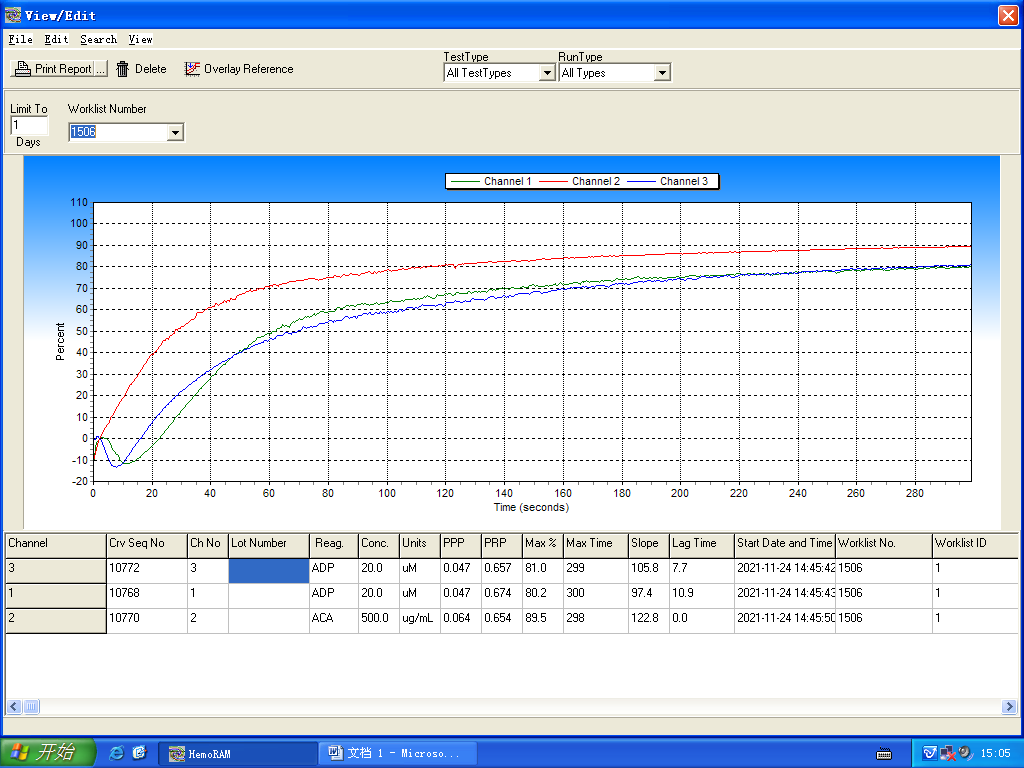


402


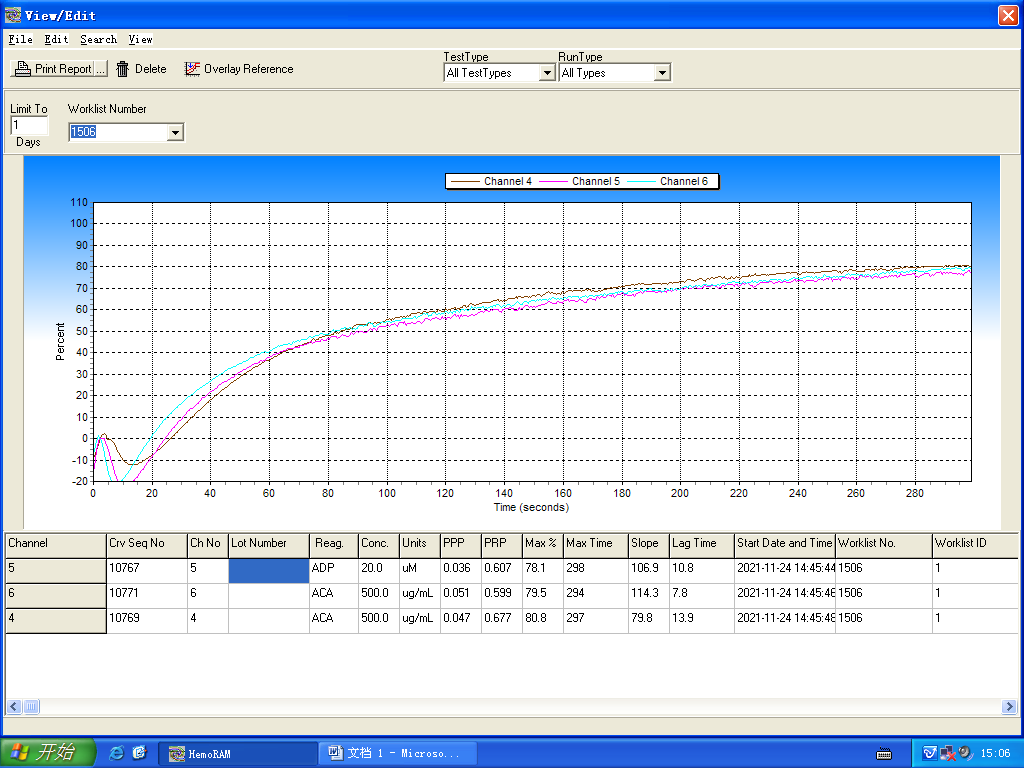


403


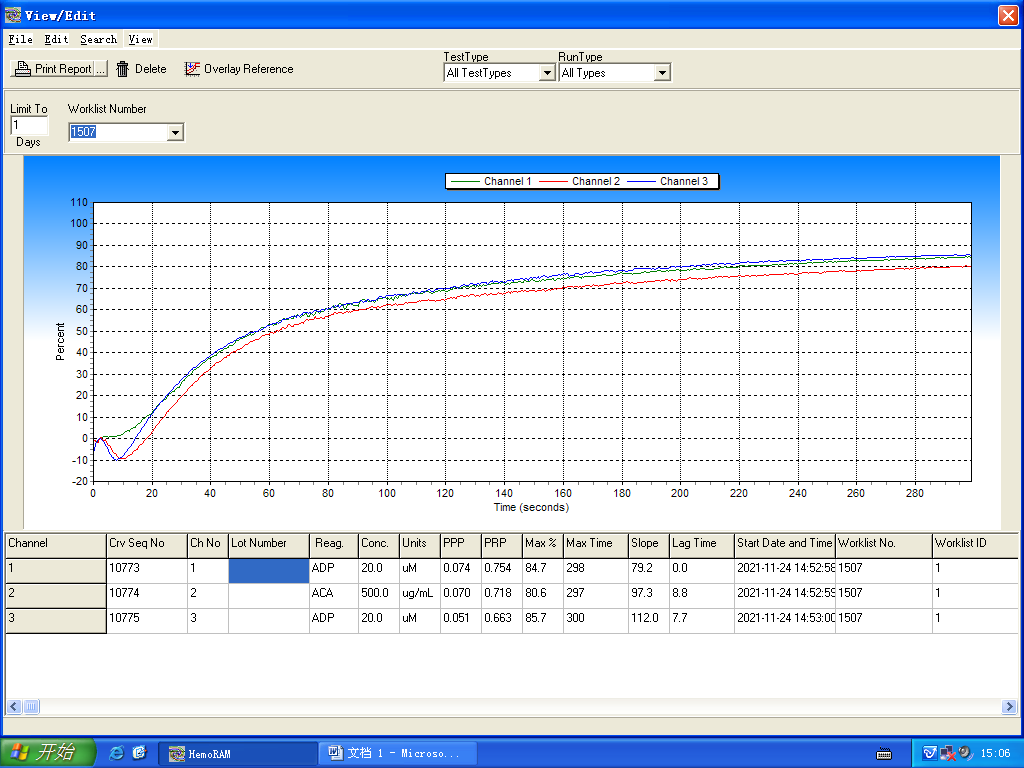

Supplement: Appendix 1—figure 1—source data 2. — (related to Appendix 1—figure 1C). [file elife-70240-app1-fig1-data2.doc]

004


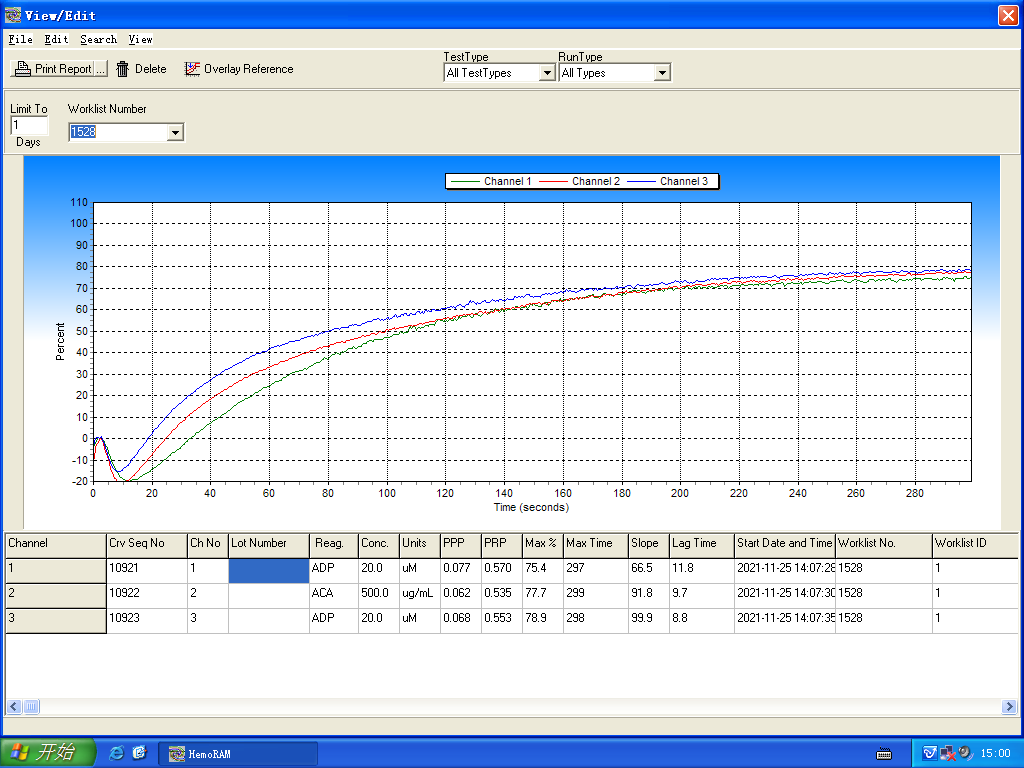


005


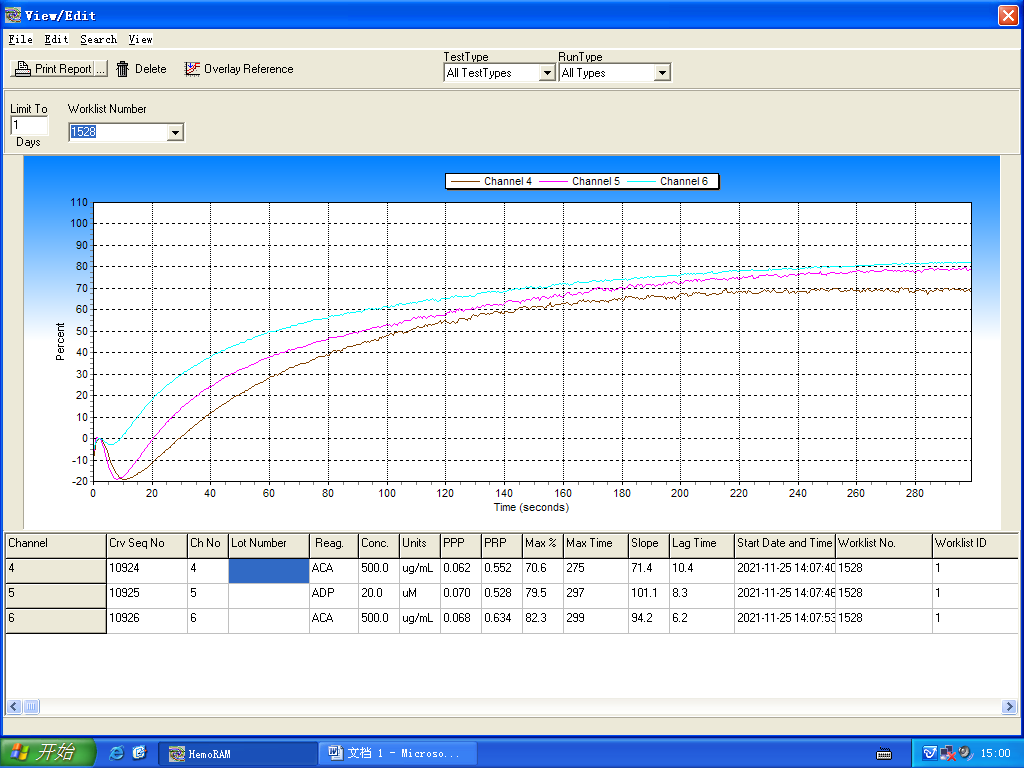


006


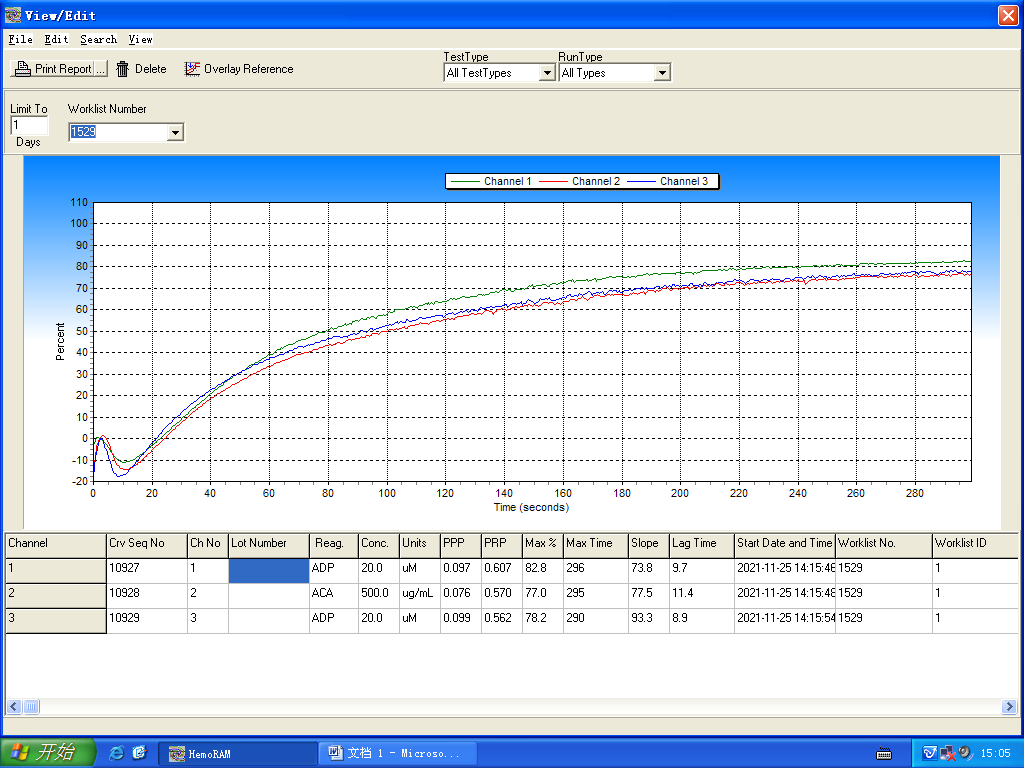


104


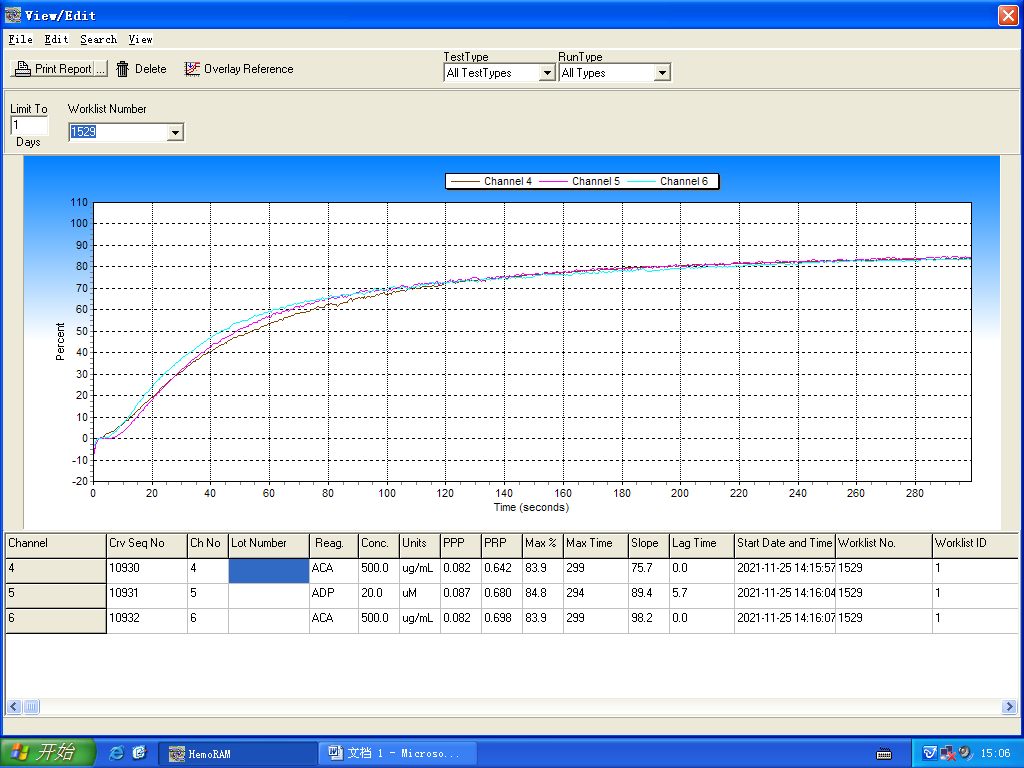


105


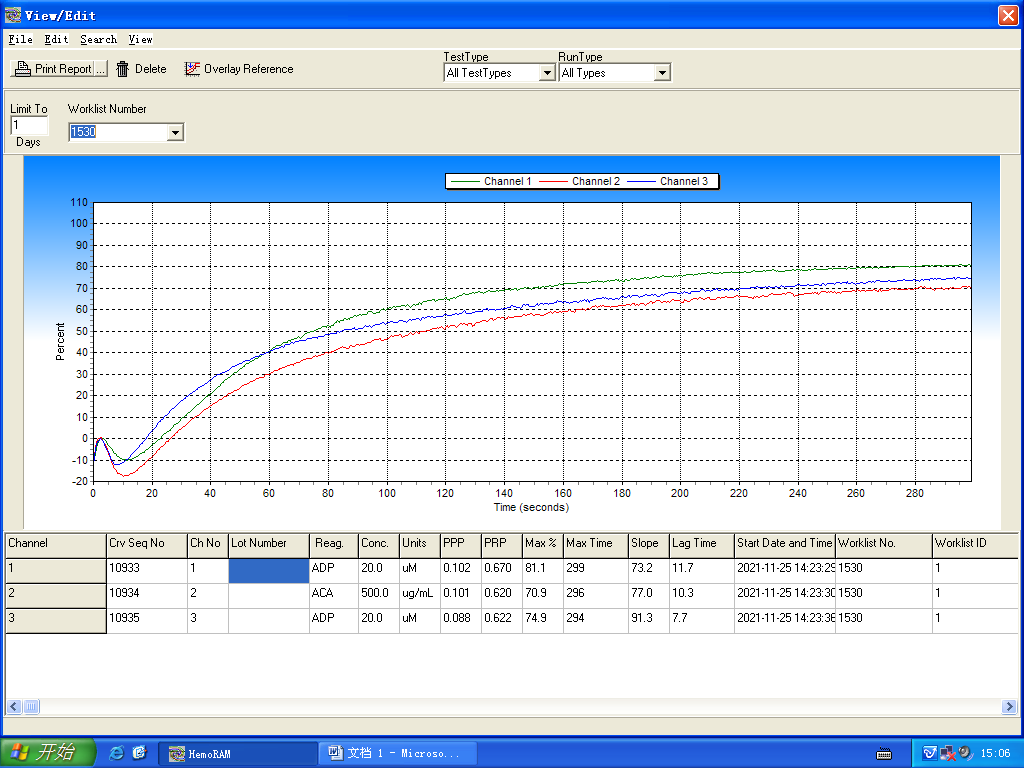


106


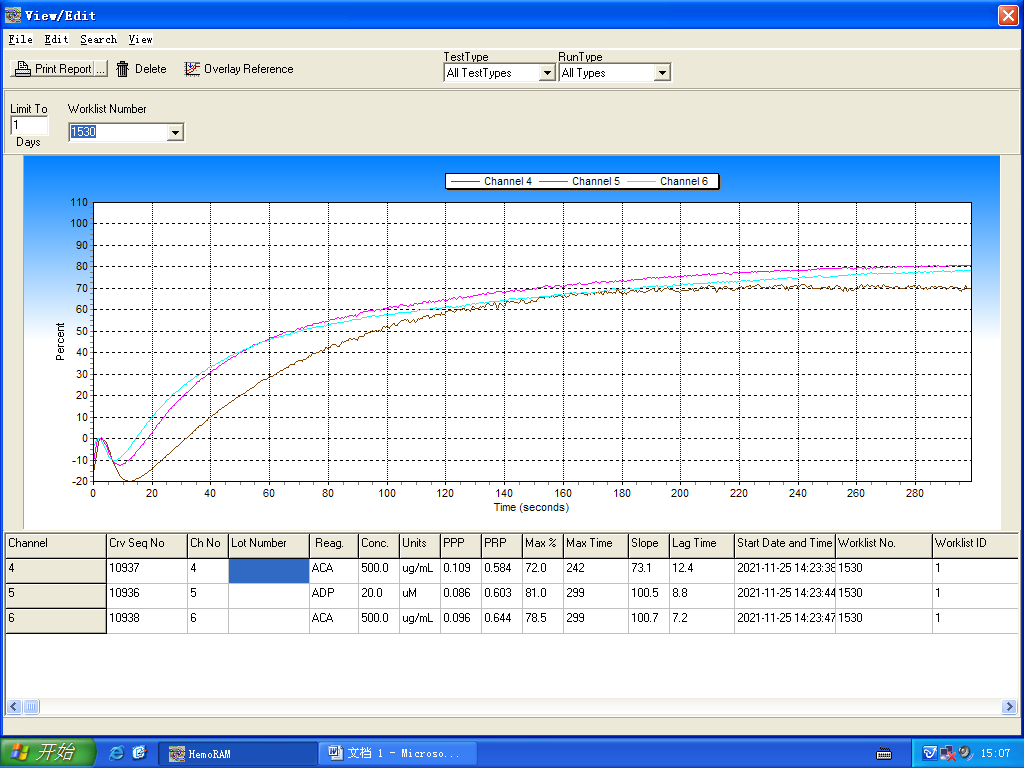


404


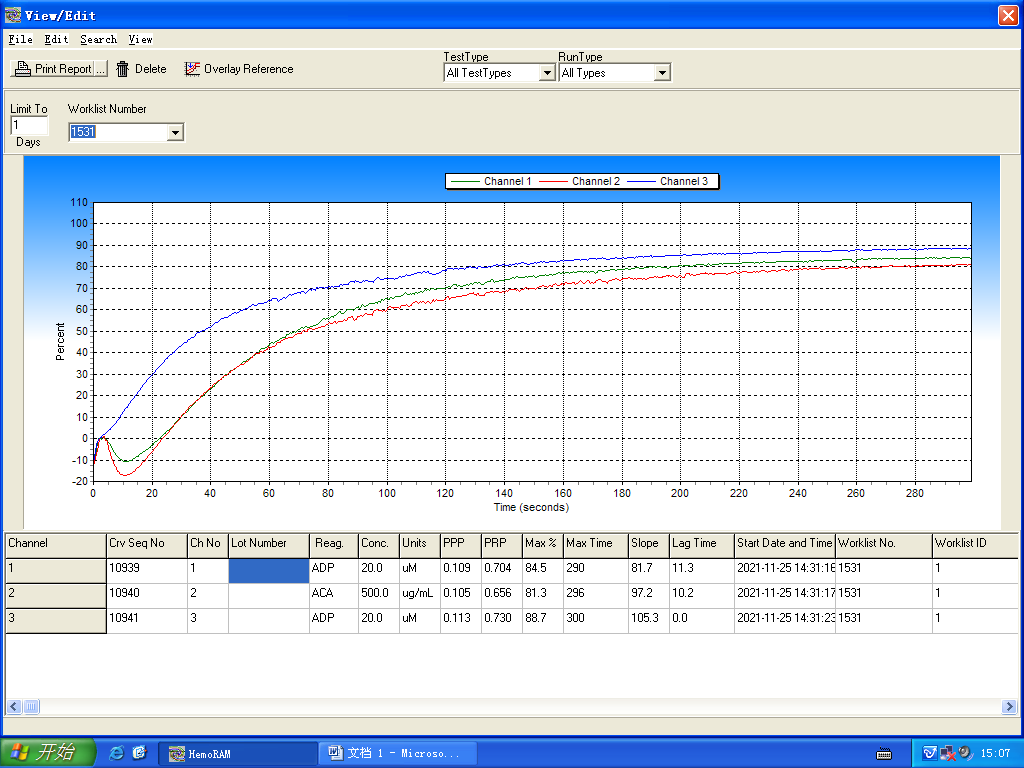


405


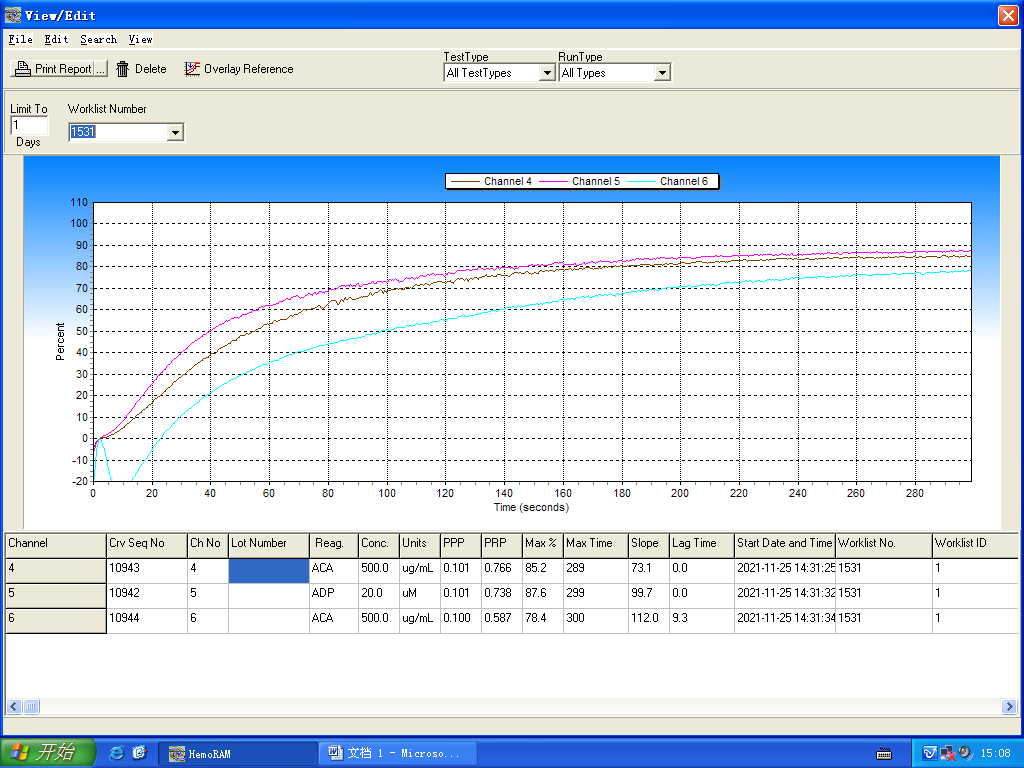


406


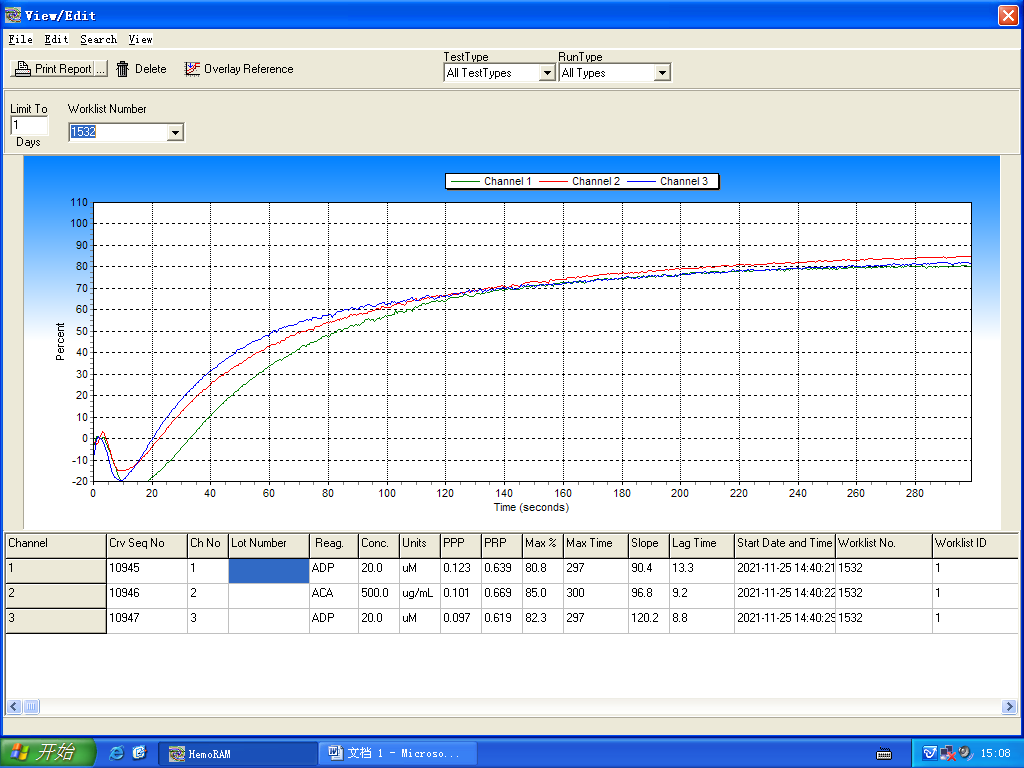

Supplement: Appendix 1—figure 1—source data 3. [file elife-70240-app1-fig1-data3.doc]
